# Putative cold acclimation pathways in *Arabidopsis thaliana *identified by a combined analysis of mRNA co-expression patterns, promoter motifs and transcription factors

**DOI:** 10.1186/1471-2164-8-304

**Published:** 2007-09-02

**Authors:** Aakash Chawade, Marcus Bräutigam, Angelica Lindlöf, Olof Olsson, Björn Olsson

**Affiliations:** 1 Department of Cell and Molecular Biology, Göteborg University, Box 462, 403 20 Göteborg, Sweden; 2 School of Humanities and Informatics, University of Skövde, Box 408, 541 28 Skövde, Sweden

## Abstract

**Background:**

With the advent of microarray technology, it has become feasible to identify virtually all genes in an organism that are induced by developmental or environmental changes. However, relying solely on gene expression data may be of limited value if the aim is to infer the underlying genetic networks. Development of computational methods to combine microarray data with other information sources is therefore necessary. Here we describe one such method.

**Results:**

By means of our method, previously published Arabidopsis microarray data from cold acclimated plants at six different time points, promoter motif sequence data extracted from ~24,000 Arabidopsis promoters and known transcription factor binding sites were combined to construct a putative genetic regulatory interaction network. The inferred network includes both previously characterised and hitherto un-described regulatory interactions between transcription factor (TF) genes and genes that encode other TFs or other proteins. Part of the obtained transcription factor regulatory network is presented here. More detailed information is available in the additional files.

**Conclusion:**

The rule-based method described here can be used to infer genetic networks by combining data from microarrays, promoter sequences and known promoter binding sites. This method should in principle be applicable to any biological system. We tested the method on the cold acclimation process in Arabidopsis and could identify a more complex putative genetic regulatory network than previously described. However, it should be noted that information on specific binding sites for individual TFs were in most cases not available. Thus, gene targets for the entire TF gene families were predicted. In addition, the networks were built solely by a bioinformatics approach and experimental verifications will be necessary for their final validation. On the other hand, since our method highlights putative novel interactions, more directed experiments could now be performed.

## Background

Plants have developed a number of different physiological and developmental responses to cope with abiotic stress. One important factor is acclimation, where mild stress conditions greatly enhance tolerance to later, more severe conditions [1]. Transcriptome analysis using microarray technology is a very powerful tool to identify cold responsive genes [2-4]. Amongst these are genes encoding transcription factors (TFs), signal transduction components, osmo-regulatory proteins, membrane stabilisation proteins, regulatory factors for protein folding, ice nucleation proteins and enzymes involved in the biosynthesis of various kinds of small molecules like polyhydroxilated sugar alcohols, amino acids and derivatives, tertiary sulphonium compounds and quaternary ammonium compounds [1,5-8]. Furthermore, molecular and genomic analyses have shown that the CBF (*C*-repeat Binding Factor) TFs have a prominent role in the cold acclimation process. However, it is known that additional pathways do exist, although they are less studied or have not even been discovered yet. Overlaps between pathways mediating cold, drought and salt stress have also been documented [4]. The plant hormone ABA, the biosynthesis of which is also induced by salt or drought stress, can be correlated to some, but not all of these pathways [9]. The only regulatory factors above *CBF *in the gene hierarchy presently known are ICE1 (Inducer of CBF Expression 1) [10], HOS1 (high expression of osmotic stress) [11] and HOS2 [12]. The signalling events that activate the *ICE1 *transcription factor gene during cold stress are not known, and the primary receptors sensing a drop in temperature ("the molecular thermometer") have not yet been characterised. Therefore, a further identification and characterization of genes involved in the molecular regulation of cold acclimation may enable us to develop plant varieties with improved tolerance to cold [1].

The development of whole-genome microarrays and the resulting availability of gene expression data has inspired many efforts to infer genetic regulatory networks using computational methods such as discrete Boolean networks [13,14], Bayesian approaches [15-17], differential equations [18], stochastic Petri nets [19,20] and clustering approaches [21]. Other approaches have explored strategies for using a combination of information sources in the network derivation process. Pilpel et al. [22] attempted to identify regulatory networks in yeast by a combinatorial analysis of promoter regions and gene expression data. Briefly, in their method, at first, for all motif pairs, all the genes containing the pair in their promoter region were identified. Then an expression coherence score was calculated for each gene cluster and significantly synergistic combinations of motifs were identified. Caselle et al. [23] developed a model to identify upstream *cis*-elements involved in gene regulation in eukaryotes. Their method grouped genes in a cluster if they shared common over-represented motifs or motif combinations in their upstream region and correlated them to gene expression. Attempts have also been made to infer regulatory networks from a combined analysis of gene expression data, promoter regions and TF binding site data [24-26]. For example, in the statistical approach developed by Xing et al. [26], transcriptional regulatory interactions were identified by analyzing 46 TFs and 658 microarray experiments on yeast gene expression at various conditions.

In this study, we apply a combined rule-based and statistical approach to infer genetic regulatory networks by integrating the information from: 1) known binding-site motifs and the corresponding TFs; 2) the time-order relationships between TFs and their target genes in terms of expression initiation; and 3) motif synergies identified by gene expression profile similarities. In our approach, genes containing known over-represented motifs are grouped into disjoint clusters fulfilling three constraints. First, all genes in each cluster must contain in their upstream regions the same known motif or combination of motifs, for which there is a known binding TF. Second, the earliest recorded time-point of significant expression of each gene in the cluster must occur at the same time as the first recorded time-point of significant expression of the gene encoding the regulating TF, or at the immediately following time-point. Third, the expression profiles of the genes in the cluster must show a higher correlation than the expression profiles of randomly selected genes. Applying these three constraints leads to formation of a grouping of the genes, based on which a regulatory network is derived by linking each known TF to the group(s) of co-expressed genes that it regulates.

Motifs or *cis*-elements are the regulatory regions found within the promoter region of any gene that controls the expression of that particular gene. Thus, in order to identify the motifs to be used for the first constraint, we searched the upstream sequences (positions -1000 to -1 bp) for the presence of known motifs using the Patmatch tool [27]. However, since the mere presence of a motif in the upstream region of a gene is not sufficient to prove its role in regulation, our method only includes a motif if its frequency of occurrence in the upstream region is greater than the frequency by which it is expected to occur by chance. For estimating the over-representation of the motifs within the 1 kb upstream region (the first constraint), the number of occurrences of the motif was calculated and the motif was considered over-represented if its number of occurrences upstream of the particular gene was found to be higher than the upper bound of the confidence interval of its average number of occurrences for all genes (see Methods). In most cases, the upper bound of the confidence interval was well below 1, which meant that a single occurrence of the motif was considered as a case of over-representation when using this method.

The second constraint was implemented by identifying whether the first time-point of significant expression of each target gene occurred at or after the first time-point of significant expression of the TF-encoding gene. Any genes not fulfilling this constraint were excluded from the cluster.

For the third constraint, we used the method of Pilpel et al. [22], where the average similarity of the expression profiles of a group of potentially co-regulated genes is compared with that of an equal number of randomly selected genes. Genes were considered to be co-regulated if their degree of similarity was significantly higher than that of the randomly selected genes.

## Results

Our method for discovery of putative genetic interactions was applied on the microarray data obtained from the cold acclimation experiments of ~24,000 genes conducted on the plant *Arabidopsis thaliana *and generated by AtGenExpress (see Acknowledgements). The processed data was downloaded from the NASC repository [28]. From this dataset, genes that were differentially expressed by at least 2.5 folds at one or more time-points were selected. Overall, 1665 genes were up-regulated and 1830 genes down-regulated. All these genes (3495 in total) were further analyzed. A number of TF-expressing genes were identified (table 1) from the up-regulated gene set and were grouped into their corresponding TF families. Those TFs for which the binding sites were experimentally determined were selected in order to identify the target genes for that corresponding TF family. When applying the here described selection criteria to the up- and the down-regulated gene sets, 670 genes in the up- and 1358 genes in the down-regulated set passed through all the criteria and were grouped into 49 and 46 clusters respectively, containing varying numbers of genes. Each individual cluster is thus predicted to be regulated by one or more TF families (table 2, 3) (see also Additional files 1, 2, 3, 4, 5, 6). Interestingly, in most of the cases, the target genes are predicted to be regulated by more than one TF. This strongly suggests that combinatorial control is very common in stress-related gene regulation. A combinatorial control of gene regulation makes a lot of sense when the stimulus involves a variety of different signals, like in cold stress. This means that relatively few TFs can control the expression of a large number of genes.

**Table 1:** Transcription factors and their binding site motifs

**TF Family^*a*^**	**Binding site^*b*^**	**CI threshold^*c*^**	**Accession^*d*^**	**Name^*e*^**	**Ref^*f*^**
**C2C2-DOF**	WAAAG	9.8	At3g50410	OBP1	[55]
			At5g60850	OBP4	
			At1g26790	-	
			At1g69570	DOF1.1	
			At5g39660	DOF5.2	
			At3g47500	DOF3.3	
**WRKY**	TTTGACY	0.7	At4g01250	WRKY22	[49]
			At2g38470	WRKY33	
			At1g80840	WRKY40	
			At2g46400	WRKY46	
			At4g23810	WRKY53	
			At4g24240	WRKY7	
			At3g56400	WRKY70	
**MYB**	AAMAATCT	0.3	At2g46830	CCA1	[56]
**AP2/EREBP**	AGCCGCC	10^-2^	At4g17500	ERF1	[57]
			At5g47230	ERF5	
**BZIP**	CACGTGG (*or*) TGACGTGG (*or*) ATGACGTCAT		At1g49720	ABRE	
		10^-2^	At3g17609	-	
		10^-2^	At2g31370	BZIP59	[58]
		10^-3^	At4g01120	GBF2	[59]
			At2g46270	BZIP55	
			At4g34590	ATB2	
**BHLH**	CANNTG	6.8	At3g05800	-	[60]
**NAC**	CATGTG	0.5	At1g01720	ATAF1	[48]
			At3g49530	-	
			At4g27410	RD26	
**MADS**	CCWWWWWWGG	0.1	At2g45660	AGL20	[61]
**AP2**	CCGAC	0.5	At4g25480	CBF3	[62]
			At4g25470	CBF1	
**TCP2**	GTGGNCCC	0.2	At4g18390	-	[63]
**HSF**	NGAANNTTCN (*or*) NTTCNNGAAN	0.8	At3g24520	-	[64]
		0.7	At4g18880	HSF21	
			At1g67970	HSF5	

Transcription factors selected for further analysis. a: Transcription factor family. b: Binding site or motif to which the TF binds, where 'W' = 'A'/'T'; 'M' = 'A'/'C'; 'Y' = 'C'/'T'; 'N' = 'A'/'T'/'G'/'C'. c: Minimum number of copies required to be found within 1 kb upstream of a gene in order for the motif to be considered as over-represented according to the confidence interval threshold. d:Locus ID of the gene. e: Common name of the TF. e: Literature reference.

**Table 2:** Motif synergy groups from up-regulated genes

**Transcription Factor(s)^*a*^**	**EC score^*b*^**	**Threshold EC score^*c*^**	**No. of genes^*d*^**
WRKY	0.22	0.18	9
TCP2	1	0.16	3
NAC, WRKY, DOF	0.27	0.2	6
NAC, HSF, WRKY	0.22	0.17	10
NAC, HSF, DOF	0.23	0.22	20
NAC, AP2(CBF), WRKY, DOF	0.25	0.17	8
NAC, AP2(CBF), WRKY	0.27	0.2	6
NAC, AP2(CBF), HSF, WRKY	0.24	0.17	13
NAC, AP2(CBF), HSF, DOF	0.18	0.17	11
NAC, AP2(CBF), HSF	0.26	0.18	25
MYB, NAC	0.56	0.17	10
MYB, MADS	1	0.16	3
MYB, DOF	0.52	0.21	34
MYB, BZIP, DOF	0.33	0.16	3
MYB, BZIP, BHLH, NAC, DOF	0.33	0.16	3
MYB, BZIP	1	0.17	4
MYB, BHLH, NAC, DOF	0.47	0.2	6
MYB, BHLH, NAC	0.36	0.17	11
MYB, BHLH, MADS	1	0.16	3
MYB, BHLH, DOF	0.33	0.18	14
MYB, BHLH	0.69	0.17	10
MYB	0.59	0.2	19
MADS, DOF	0.8	0.2	5
MADS	1	0.17	8
HSF, WRKY, DOF	0.36	0.17	8
HSF, WRKY	0.22	0.18	9
HSF	0.21	0.2	28
DOF	0.23	0.19	122
BZIP, WRKY, DOF	0.33	0.17	4
BZIP, NAC, HSF	0.33	0.16	3
BZIP, NAC, AP2(CBF), HSF, WRKY, DOF	0.33	0.16	3
BZIP, NAC, AP2(CBF), HSF	0.33	0.16	3
BZIP, NAC, AP2(CBF), DOF	0.4	0.2	5
BZIP, NAC, AP2(CBF)	0.67	0.16	3
BZIP, DOF	0.22	0.18	9
BZIP, BHLH, NAC, DOF	0.67	0.16	3
BZIP, BHLH	0.44	0.18	9
BZIP	0.52	0.21	33
BHLH, NAC, DOF	0.27	0.22	21
BHLH, NAC	0.38	0.2	19
BHLH, MADS, DOF	1	0.16	3
BHLH, DOF	0.49	0.21	34
BHLH	0.45	0.21	35
AP2(CBF), HSF, DOF	0.19	0.18	16
AP2(CBF), HSF	0.23	0.2	32
AP2(CBF), DOF	0.27	0.17	11
AP2(CBF)	0.27	0.2	6
AP2(AtERF), HSF, WRKY	0.33	0.16	3
AP2(AtERF), DOF	0.5	0.17	4

Results obtained by applying the method using the confidence interval criterion on the genes filtered from the 24 k gene array data set. a: TF families. b: EC score obtained from the cluster of putative target genes predicted by this method. c: Threshold above which the EC score is acceptable. d: Number of putative target genes that are regulated by the TF families that are indicated in the first column.

**Table 3:** Motif synergy groups from down-regulated genes

**Regulators**	**EC score**	**Threshold EC score**	**No. of genes**
AP2(CBF)	0.34	0.22	55
AP2(CBF), HSF	0.42	0.21	34
BHLH	0.58	0.20	214
BHLH, AP2(CBF)	0.51	0.21	30
BHLH, AP2(CBF), HSF	0.23	0.20	19
BHLH, HSF	0.27	0.21	33
BHLH, MADS	0.50	0.20	5
BHLH, MADS, AP2(CBF)	1.00	0.16	3
BHLH, TCP2	0.85	0.16	8
BZIP	0.36	0.18	9
BZIP, BHLH, AP2(CBF)	0.53	0.20	6
BZIP, HSF	0.20	0.20	6
DOF	0.38	0.20	154
DOF, AP2(CBF)	0.28	0.22	65
DOF, BHLH	0.37	0.19	119
DOF, BHLH, AP2(CBF)	0.25	0.20	27
DOF, BHLH, AP2(CBF), HSF	0.28	0.22	20
DOF, BHLH, HSF	0.31	0.22	43
DOF, BHLH, MADS	0.39	0.17	8
DOF, BZIP	0.33	0.16	3
DOF, BZIP, AP2(CBF)	0.40	0.20	6
DOF, BZIP, BHLH, HSF	0.53	0.20	6
DOF, BZIP, HSF	0.52	0.20	7
DOF, HSF	0.21	0.21	60
DOF, MADS	1.00	0.16	3
DOF, MYB	0.54	0.20	35
DOF, MYB, AP2(CBF)	0.30	0.20	5
DOF, MYB, BHLH	0.47	0.18	20
DOF, MYB, MADS	0.33	0.17	4
DOF, WRKY	0.26	0.21	32
DOF, WRKY, AP2(CBF)	0.25	0.20	18
DOF, WRKY, BHLH	0.24	0.20	25
DOF, WRKY, BHLH, AP2(CBF), HSF	0.33	0.18	15
DOF, WRKY, BHLH, HSF	0.21	0.20	27
DOF, WRKY, HSF	0.26	0.22	45
MADS	0.80	0.20	6
MYB	0.57	0.18	20
MYB, AP2(CBF)	0.57	0.17	8
MYB, BHLH	0.63	0.18	14
WRKY	0.26	0.20	28
WRKY, AP2(CBF), HSF	0.21	0.18	17
WRKY, BHLH	0.40	0.20	27
WRKY, BHLH, AP2(CBF)	0.25	0.18	17
WRKY, BHLH, HSF	0.20	0.20	25
WRKY, BZIP, HSF	0.33	0.17	4
WRKY, HSF	0.25	0.20	23

Motif synergy groups for down-regulated genes. For explanations see legend in table 2.

To our knowledge, no systematic study has previously been done on combinatorial control of stress signalling in *Arabidopsis*. In our computational approach we therefore identified several previously unknown combinations of different TFs regulating a cluster of target genes (table 2 and 3). For example, HSFs (Heat Shock Factor) is identified as the sole family of regulators of 28 up-regulated genes. However, in total, HSFs are predicted to regulate 184 genes. Thus, the remaining 156 genes (85%) seem to be under combinatorial control of the HSF and other TF families, such as BZIP, AP2 (AtERF and CBF), WRKY, DOF and NAC. Similar results could be observed with the other clusters of genes, which strongly indicates that cold-regulation at the molecular level can be more efficiently studied if combinatorial regulation is taken into consideration.

One way to computationally validate combinatorial regulation is through expression coherence scores. This technique was developed by Pilpel *et al*. [22] in order to study the effect on gene expression patterns when adding or subtracting motif combinations that seem to be directly responsible for particular expression patterns. For each motif or motif combination, Pilpel *et al*. calculated the expression coherence (EC) score, a measure of similarity of the expression profiles of all the genes containing that motif only or in combination with other motifs. Gene clusters were accepted if their EC scores were found to be significant. We applied this technique to the obtained results, but with some modifications. In our work, the genes were clustered together only if they were predicted to be regulated by the same set of TFs. Thereafter, the EC scores were calculated for all gene clusters and a particular cluster was accepted if its EC score was found to be above a certain threshold (see Methods).

**Figure 1:**
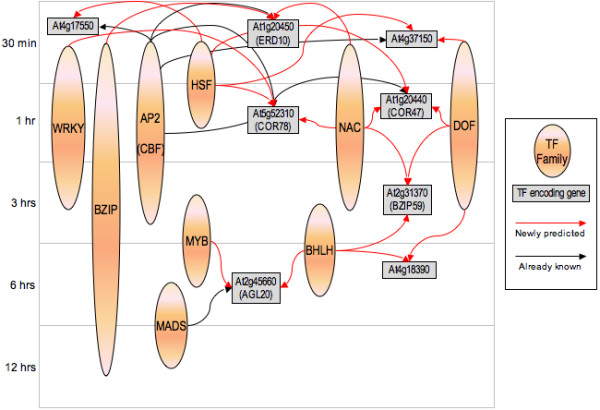
**Part of the predicted regulatory network**. Regulatory network at the gene family level. The origin of the arrow indicates the regulating TF family and the endpoint of the arrow indicates the target gene. The time scale is shown on the vertical axis.

**Figure 2:**
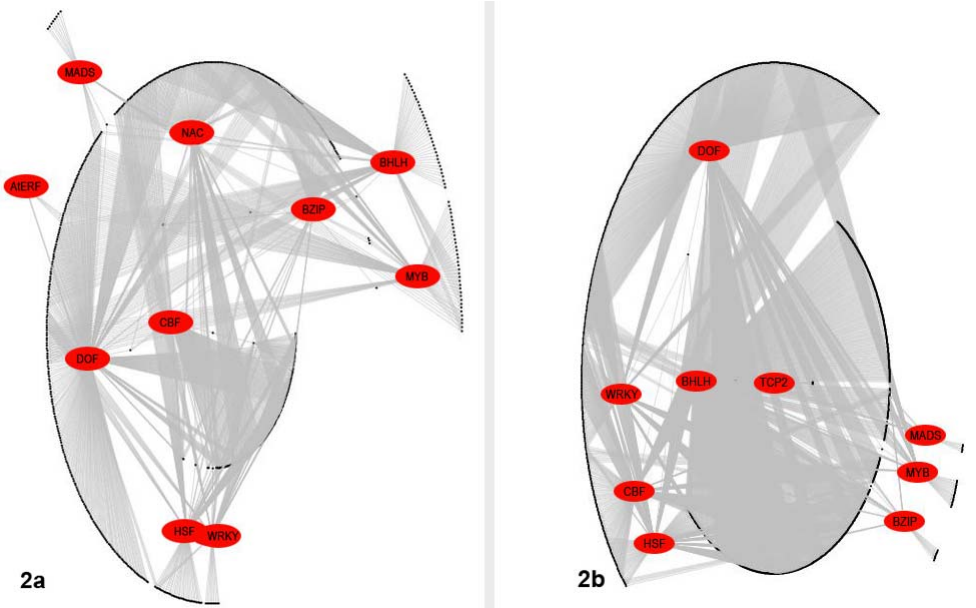
**Large scale regulatory network**. Overview of the regulatory network. 2a. Up-regulated gene network. 2b. Down-regulated gene network. The entries in red ovals represent the TFs and the black dots represent target genes. The networks were generated using Graphviz version 1.13–16 [65].

The putative regulatory networks (fig 1 and 2) generated by this approach again demonstrate the degree of complexity that seems to be involved in the regulation of the target genes. As seen in figure 1, the method predicts that TFs typically co-operate with several different TFs to regulate the downstream target gene(s). Figure 2 gives an overview of the predicted regulation of the analyzed genes. In general, most of the TFs regulate a core cluster of genes that are not under combinatorial control and in addition also pair up with other TFs in different combinations to regulate an entirely different cluster of genes. This complex pattern of activity may be required to accomplish the large and diverse set of morphological and physiological changes that are necessary to resist cold.

For each TF family, all of its putative targets were grouped and for each such group of genes, the functional annotations were analysed. The statistical significance of the annotations was estimated online with the MIPS web interface [29]. From this analysis, it can be deduced that different TF families preferentially regulate different cellular processes involved in cold regulation. For instance, in the up-regulated set, the cell rescue, defence and virulence process is predicted to be preferentially regulated by the proteins belonging to the AP2 family (CBFs; *p *≤ 10^-4^); the transcription process by the WRKY (*p *≤ 10^-4^), DOF (*p *≤ 10^-4^) and NAC (*p *≤ 10^-3^) families; metabolic processes by the BHLH family (*p *≤ 10^-2^) and energy processes by the BZIP family (*p *≤ 10^-3^) (table 4). However, there are also instances where two or more families of TFs have a significant score for the same biological process. This pattern suggests that when required, many families of TFs participate in combinatorial regulation to regulate more complex biological processes. This is also supported by the work of by Tong et al. [30] where they found that in yeast, synthetic genetic relationships frequently coincide with a known functional relationship between gene pairs.

**Table 4:** Functional annotations of the TF targets

**MIPS Bincode^*a*^**	**MIPS Annotation category^*b*^**	**BHLH^*c*^**	**BZIP^*d*^**	**HSF^*e*^**	**NAC^*f*^**	**WRKY^*g*^**	**AP2 (CBF)^*h*^**	**DOF^*i*^**	**MYB^*j*^**	**MADS^*k*^**	**TCP2^*l*^**	**AP2 (AtERF)^*m*^**
**01**	**METABOLISM**	**10^-2^**	10^-1^	10^-1^	10^1^	10^1^	10^-1^	10^-1^	10^-1^	10^-1^	10^-1^	10^1^
01.01.03	Assimilation of ammonia, metabolism of the glutamate group	**10^-2^**	N/A	N/A	N/A	N/A	N/A	10^-1^	10^-1^	N/A	N/A	N/A
01.03.04.03	Pyrimidine nucleotide anabolism	**10^-2^**	N/A	N/A	N/A	N/A	N/A	10^-1^	N/A	N/A	N/A	N/A
01.05	C-compound and carbohydrate metabolism	10^-1^	**10^-2^**	10^-1^	10^1^	10^-1^	10^1^	**10^-2^**	10^-1^	10^-1^	N/A	N/A
01.05.01.03	C-compound, carbohydrate anabolism	N/A	N/A	N/A	N/A	N/A	N/A	N/A	N/A	**10^-2^**	N/A	N/A
01.05.01.03.02	Polysaccharide biosynthesis	N/A	N/A	N/A	N/A	N/A	N/A	N/A	N/A	**10^-2^**	N/A	N/A
01.06	Lipid, fatty acid and isoprenoid metabolism	10^-1^	10^1^	N/A	10^-1^	N/A	10^-1^	10^-1^	10^1^	N/A	**10^-2^**	N/A
01.06.01.01	Phospholipid biosynthesis	N/A	**10^-2^**	N/A	**10^-2^**	N/A	**10^-2^**	10^1^	N/A	N/A	N/A	N/A
01.06.04	Degradation of lipids, fatty acids and isoprenoids	**10^-3^**	N/A	N/A	10^-1^	N/A	N/A	**10^-2^**	N/A	N/A	**10^-2^**	N/A
01.20.17	Biosynthesis of secondary products derived from primary amino acids	**10^-3^**	N/A	10^-1^	10^-1^	N/A	N/A	10^-1^	**10^-2^**	N/A	N/A	N/A
01.20.17.01	Biosynthesis of nonprotein amino acids	10^-1^	N/A	N/A	N/A	N/A	N/A	10^-1^	**10^-2^**	N/A	N/A	N/A
01.20.17.09	Biosynthesis of alkaloids	10^-1^	N/A	N/A	10^-1^	N/A	N/A	10^-1^	**10^-2^**	N/A	N/A	N/A
01.20.35	Biosynthesis of secondary products derived from L-phenylalanine and L-tyrosine	N/A	N/A	10^-1^	N/A	10^-1^	10^-1^	10^1^	N/A	N/A	N/A	**10^-2^**
01.20.35.01	Biosynthesis of phenylpropanoids	N/A	N/A	10^-1^	N/A	10^-1^	10^-1^	10^1^	N/A	N/A	N/A	**10^-2^**
**02**	**ENERGY**	10^-1^	**10^-3^**	**10^-2^**	10^1^	10^1^	10^-1^	**10^-2^**	10^-1^	N/A	N/A	N/A
02.13	Respiration	10^-1^	**10^-2^**	N/A	10^-1^	N/A	N/A	10^-1^	10^-1^	N/A	N/A	N/A
02.13.03	Aerobic respiration	10^-1^	10^-1^	N/A	10^-1^	N/A	N/A	**10^-2^**	N/A	N/A	N/A	N/A
02.19	Metabolism of energy reserves (e.g. glycogen, trehalose)	10^-1^	**10^-4^**	**10^-2^**	10^-1^	N/A	N/A	10^-1^	N/A	N/A	N/A	N/A
**04**	**STORAGE PROTEIN**	N/A	N/A	N/A	N/A	N/A	N/A	10^-1^	N/A	N/A	N/A	**10^-2^**
**10.01**	**DNA processing**	10^-1^	**10^-2^**	10^1^	N/A	10^-1^	N/A	10^-1^	N/A	N/A	N/A	N/A
10.03.04.03	Chromosome condensation	10^-1^	**10^-2^**	N/A	N/A	N/A	N/A	N/A	N/A	N/A	N/A	N/A
**11**	**TRANSCRIPTION**	10^-1^	**10^-2^**	**10^-2^**	**10^-3^**	**10^-4^**	10^-1^	**10^-4^**	10^1^	10^-1^	N/A	10^-1^
11.02	RNA synthesis	10^-1^	**10^-2^**	**10^-2^**	**10^-4^**	**10^-5^**	10^-1^	**10^-4^**	10^-1^	10^-1^	N/A	10^-1^
11.02.01	rRNA synthesis	N/A	N/A	**10^-2^**	10^-1^	10^-1^	**10^-2^**	N/A	N/A	N/A	N/A	N/A
11.02.03	mRNA synthesis	10^-1^	**10^-2^**	**10^-2^**	**10^-3^**	**10^-4^**	10^-1^	**10^-4^**	10^-1^	10^-1^	N/A	10^-1^
11.02.03.04	Transcriptional control	10^-1^	**10^-2^**	10^-1^	**10^-3^**	**10^-3^**	10^-1^	**10^-3^**	10^1^	10^-1^	N/A	10^-1^
11.04	RNA processing	10^1^	10^-1^	**10^-3^**	**10^-2^**	**10^-2^**	**10^-2^**	10^-1^	10^-1^	N/A	N/A	N/A
11.04.01	rRNA processing	N/A	N/A	**10^-3^**	10^-1^	**10^-2^**	**10^-3^**	N/A	N/A	N/A	N/A	N/A
11.04.02	tRNA processing	N/A	**10^-2^**	N/A	N/A	N/A	N/A	10^-1^	N/A	N/A	N/A	N/A
11.04.03	mRNA processing (splicing, 5'-, 3'-end processing)	10^-1^	N/A	**10^-2^**	**10^-2^**	10^-1^	10^-1^	10^-1^	10^-1^	N/A	N/A	N/A
11.04.03.01	Splicing	10^-1^	N/A	10^-1^	**10^-2^**	10^-1^	10^-1^	10^-1^	**10^-2^**	N/A	N/A	N/A
**12.04.03**	**Translation termination**	**10^-2^**	N/A	N/A	**10^-2^**	N/A	N/A	N/A	N/A	N/A	N/A	N/A
**16.03.01**	**DNA binding**	10^-1^	**10^-2^**	N/A	N/A	N/A	N/A	N/A	N/A	N/A	N/A	N/A
16.13	C-compound binding	N/A	N/A	10^-1^	N/A	**10^-2^**	N/A	10^-1^	N/A	N/A	N/A	N/A
**18**	**PROTEIN ACTIVITY REGULATION**	N/A	10^-1^	10^-1^	10^-1^	**10^-2^**	10^-1^	10^-1^	N/A	N/A	N/A	N/A
18.01.01	Modification	N/A	N/A	10^-1^	N/A	**10^-2^**	N/A	10^-1^	N/A	N/A	N/A	N/A
18.02.05	Regulator of G-protein signalling	N/A	**10^-2^**	N/A	**10^-2^**	**10^-2^**	**10^-2^**	10^-1^	N/A	N/A	N/A	N/A
**20**	**CELLULAR TRANSPORT, TRANSPORT FACILITATION AND TRANSPORT ROUTES**	**10^-2^**	10^1^	10^1^	10^-1^	N/A	10^1^	10^-1^	10^-1^	10^-1^	N/A	N/A
20.01	Transported compounds (substrates)	**10^-2^**	10^1^	10^1^	10^-1^	N/A	10^-1^	**10^-2^**	10^-1^	N/A	N/A	N/A
20.01.01	Ion transport	10^-1^	N/A	10^1^	10^1^	N/A	10^-1^	**10^-2^**	10^-1^	N/A	N/A	N/A
20.01.03	C-compound and carbohydrate transport	10^-1^	N/A	10^-1^	**10^-2^**	N/A	**10^-2^**	N/A	10^-1^	N/A	N/A	N/A
20.09.07	Vesicular transport (Golgi network, etc.)	**10^-2^**	10^-1^	N/A	10^-1^	N/A	N/A	10^-1^	N/A	10^-1^	N/A	N/A
**30.05.01.10**	**Two-component signal transduction system (sensor kinase component)**	N/A	**10^-2^**	N/A	N/A	N/A	N/A	N/A	N/A	N/A	N/A	N/A
**32**	**CELL RESCUE, DEFENSE AND VIRULENCE**	10^-1^	10^1^	**10^-2^**	**10^-2^**	**10^-2^**	**10^-4^**	**10^-2^**	10^-1^	N/A	N/A	10^-1^
32.01	Stress response	**10^-2^**	10^1^	**10^-3^**	**10^-2^**	10^1^	**10^-4^**	**10^-2^**	10^-1^	N/A	N/A	10^-1^
32.05	disease, virulence and defense	10^1^	10^1^	10^1^	10^-1^	**10^-2^**	10^-1^	10^-1^	10^-1^	N/A	N/A	N/A
32.05.03	defense related proteins	N/A	10^-1^	10^-1^	10^-1^	**10^-3^**	**10^-3^**	10^-1^	10^-1^	N/A	N/A	N/A
**36.25.16**	**Immune response**	N/A	N/A	10^-1^	N/A	**10^-2^**	N/A	10^-1^	N/A	N/A	N/A	N/A
**40**	**CELL FATE**	**10^-2^**	10^1^	N/A	10^1^	N/A	10^-1^	10^-1^	10^1^	N/A	N/A	N/A
40.20	Cell aging	**10^-3^**	N/A	N/A	N/A	N/A	N/A	N/A	N/A	N/A	N/A	N/A
**41.05.10**	**Late embryonic development**	N/A	N/A	N/A	**10^-2^**	N/A	**10^-2^**	N/A	N/A	N/A	N/A	N/A
**42.02**	**Eukaryotic plasma membrane**	N/A	**10^-2^**	N/A	10^-1^	**10^-2^**	10^-1^	N/A	N/A	N/A	N/A	N/A
42.10	Nucleus	10^-1^	**10^-2^**	N/A	N/A	N/A	N/A	N/A	N/A	N/A	N/A	N/A
42.10.03	Organization of chromosome structure	10^-1^	**10^-2^**	N/A	N/A	N/A	N/A	N/A	N/A	N/A	N/A	N/A
**70**	**SUBCELLULAR LOCALIZATION**	**10^-2^**	**10^-2^**	10^-1^	10^1^	10^-1^	10^1^	10^-1^	10^1^	10^1^	N/A	10^-1^
70.01	Cell wall	10^-1^	N/A	N/A	**10^-2^**	10^-1^	10^-1^	10^-1^	N/A	N/A	N/A	N/A
70.08	Golgi	10^-1^	N/A	N/A	N/A	N/A	N/A	10^-1^	N/A	**10^-2^**	N/A	N/A
70.09	Intracellular transport vesicles	**10^-2^**	10^-1^	N/A	10^-1^	N/A	N/A	**10^-2^**	N/A	N/A	N/A	N/A
70.10	Nucleus	10^1^	**10^-3^**	10^-1^	10^1^	**10^-2^**	10^-1^	10^1^	N/A	N/A	N/A	10^-1^
70.10.03	Chromosome	10^-1^	**10^-2^**	N/A	N/A	N/A	N/A	N/A	N/A	N/A	N/A	N/A
**73.03.09**	**Immune cell**	N/A	N/A	**10^-2^**	N/A	**10^-2^**	N/A	**10^-2^**	N/A	N/A	N/A	N/A
**98**	**CLASSIFICATION NOT YET CLEAR-CUT**	10^-1^	**10^-3^**	**10^-3^**	**10^-3^**	**10^-2^**	**10^-3^**	**10^-4^**	10^-1^	10^-1^	10^-1^	10^-1^

Significant functional annotations of the cluster of genes putatively regulated by the TF-encoding genes. a and b: annotation categories in the MIPS functional annotation database. c to m: *p*-values obtained for all the genes putatively regulated by the corresponding TF. The significance of the annotation (*p*) for the list of all target genes putatively regulated by a TF was calculated using the MIPS online interface. Entries in bold denote significant annotations (*p *< 0.05).

## Discussion

The availability of both genomic and microarray data in several model organisms has opened up possibilities to elucidate important genetic regulatory mechanisms in these models. Here we made an attempt to expand the known genetic network underlying the cold acclimation process in *Arabidopsis *by a systematic integration of genomic and transcriptome data. Our approach was able to correctly identify earlier described targets from the literature. Besides identifying new potential targets of the known transcription factors, our approach also identified putative novel pathways in the cold acclimation process. Thus, we illuminated pathways regulated by the BHLH, BZIP, HSF, AP2, NAC, WRKY, DOF, MADS, MYB, and TCP2 families of TF-genes.

The BHLH family of TFs is known to be involved in anthocyanin biosynthesis, light response, flower development and abiotic stress [31]. Analysis of up-regulated targets indicates that under cold stress there are 171 up-regulated target genes of BHLH, several of which are connected to metabolism, transport activities, stress response and cell fate activities (*p *≤ 0.05) (table 4). One important response in cold acclimation is starch degradation. Two especially important genes in this process are *phosphoglycerate mutase *(*PGM*) (*At1g09780*) and *pyruvate kinase *(*PK*) (*At5g56350*), which encode enzymes that take part in glycolysis and gluconeogenesis in the first steps of the starch biosynthesis pathway. Since sugar levels have to be closely regulated during cold acclimation, *PGM *is therefore an important target gene. BHLH TFs are known to regulate the expression levels of *PGM *[32] and *PK *[33], both of which were correctly identified by our approach. In addition, the MYB, NAC and DOF TFs were also predicted to be regulating *PGM*, while *PK *was predicted to be regulated by *MYB*. These are to our knowledge all novel interactions. Moreover, *Adenosylhomocysteinase *(*AHC*) (*At3g23810*), a gene involved in methionine biosynthesis, was also identified as being regulated by BHLH and DOF. This could be a novel interaction, since no evidence relating *AHC *to BHLH and DOF could be found in the literature. On the other hand, it is known that *alcohol dehydrogenase *(*ADH1*) (*At1g77120*) catalyzes the inter-conversion of aldehyde and alcohol. Abe et al. [34] found that transgenic plants over-expressing *AtMYC2 *(*BHLH*) and *AtMYB2 *(*MYB*) produced elevated levels of *ADH1*, while knockout mutants of *AtMYC2 *were less sensitive to ABA and showed significantly decreased expression of *ADH1 *[34]. Thus, Abe et al. [34] suggested that both MYC2 and MYB2 TFs are involved in ABA-regulated gene expression of *ADH1 *under drought and salt stress and that BHLH TFs putatively regulate *ADH*. Our model correctly predicted BHLH and MYB TF family genes as regulating *ADH1*. Dolferus et al. [35] showed that mutation of the G-box motif located between -216 and -209 upstream of *ADH *significantly reduced induction of *ADH *specifically under cold stress but did not affect expression under un-induced conditions. As BZIPs are known to bind to the G-box motif, our approach correctly predicted BZIP as a regulating factor of *ADH1*. In addition, DOF TFs were also predicted to regulate *ADH1 *expression. This is intriguing; since to our knowledge, no literature evidence is available that demonstrates the regulation of *ADH1 *by *DOF *TFs.

The BZIP family of TFs is known to be involved in seed-storage, gene expression, photomorphogenesis, leaf development, flower development, defence response, ABA response, gibberellin biosynthesis [31] and cold stress [36,37]. An analysis of the functional annotations of the up-regulated target genes showed that processes regulated by BZIP TFs include energy (*p *≤ 10^-3^) and in particular transcription (*p *≤ 10^-2^) (table 4). In the cold acclimated plant cells, sucrose is produced in large quantities. The source of sucrose that accumulates during cold acclimation is not known, but it could be produced from starch degradation [38-41]. The enzyme *α*-amylase is involved in the starch degradation pathway and transcript levels of the *α*-amylase gene are induced (>2.5 folds) during cold acclimation. Yamauchi suggested that BZIP regulates *α*-amylase gene expression [42]. In accordance with this hypothesis, our approach identified BZIP as a putative key regulator of *α-amylase *(*At1g69830*).

Under heat stress, HSFs are the primary molecules involved in transcriptional regulation of heat shock response [43]. The fact that *HSFs *are induced by diverse types of stresses suggests that they might play a central role in regulating the cell repair process and to counteract cytotoxic effects of protein denaturation [43,44]. Analysis of the functional annotations of the putative up-regulated HSF target genes indicates that HSFs significantly regulate biological processes such as energy (*p *≤ 10^-2^) and transcription (*p *≤ 10^-2^), as well as cell rescue, defence and virulence (*p *≤ 10^-2^) (table 4). Some of the putative targets of HSFs are involved in regulating glycolysis, glyoxylate metabolism, sucrose metabolism and trehalose biosynthesis. Trehalose 6 phosphate phosphatase (T6PP) converts trehalose 6 phosphate to trehalose. It has been previously shown that heat shock proteins and trehalose together confer thermotolerance in *Saccharomyces cerevisiae *[45]. Our model identified T6PP (*At2g22190*) as being regulated by BZIPs.

AP2 domain containing proteins are known to be involved in regulating several developmental processes including flower development, cell proliferation, secondary metabolism, abiotic and biotic stress responses, ABA response and ethylene response [8,31]. The *CBF *regulon is considered to have a major role in regulating the cold acclimation process in *A. thaliana *[1,2,8]. Analysis of functional annotations of the up-regulated set suggests that CBF TFs, as expected, significantly regulate several biological processes, including cell rescue, defence and virulence (*p *≤ 10^-4^) (table 4). Gilmour et al. [46] suggested a list of 31 genes involved in the CBF regulon. Of these, 17 genes were up-regulated by at-least 2.5 folds in the data we analyzed. Out of 17 genes, 13 contain at least one CRT motif ("CCGAC") within the 1 kb upstream region. Of these 13 genes, our approach predicted 5 genes to be regulated by CBF TFs [see Additional file 6]. In addition, TFs from other families, including BZIP, NAC, HSF and DOF, were also identified as key regulators of one or more of these target genes. In total, 142 genes were predicted to be regulated by CBF TFs.

NAC TFs are specific to plants and they are known to play a role in both developmental processes [47] and stress response [48]. Our results support the involvement of *NAC *genes in phospholipid biosynthesis (*p *≤ 10^-2^), transcription (*p *≤ 10^-3^), cell rescue, defence and virulence (*p *≤ 10^-2^) and late embryonic development (*p *≤ 10^-2^) (table 4).

WRKYs are known to be involved in several different defence responses [49]. Our analysis suggests that they are significantly involved in transcription (*p *≤ 10^-4^), protein activity regulation (*p *≤ 10^-2^), cell rescue, defence and virulence (*p *≤ 10^-2^) and in immune response (*p *≤ 10^-2^) (table 4).

Finally,*DOF *genes encode DOF domain proteins that act as transcriptional regulators in plant growth and development including seed germination, endosperm-specific expression and carbon metabolism [31]. Our analysis suggests that DOFs are significantly involved in processes such as energy (*p *≤ 10^-2^), transcription (*p *≤ 10^-4^) and cell rescue, defence and virulence (*p *≤ 10^-2^) (table 4). They also interact with transcription factors from other families, including BZIP, HSF, WRKY and NAC. Combinatorial regulation of targets by both DOF and BZIP TFs, as predicted by our model, is supported by previous work [50].

**Figure 3:**
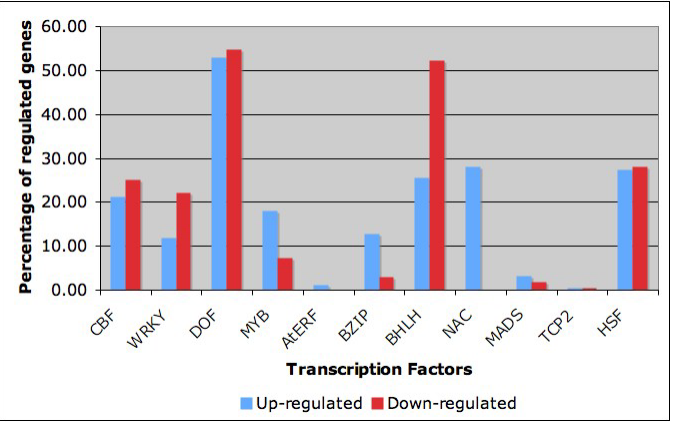
**Regulation pattern in up-regulated and down-regulated genes**. Percentage of up-regulated and down-regulated genes that are predicted to be regulated by different TF families.

From fig 3, it could be suggested that some TF families preferentially regulate either up-regulated or down-regulated genes. For example, the BHLH family of TF putatively regulates a larger number of down- than up-regulated genes. On the contrary, TFs of the MYB family of TF putatively regulates more up- than down-regulated genes. The CBF TFs, on the other hand, seem to regulate approximately equal numbers of up- and down-regulated genes.

In this work we analyzed only those motifs that were previously known from the literature. However, the approach is general and can easily be extended by using motifs that are generated by other motif finding tools. This approach is not yet capable of predicting auto-regulation, i.e., a gene regulating its own expression, or feed-back loops, and efforts will be made in the near future to find appropriate solutions for these two situations. An additional limitation is that gene targets were predicted for the TF gene families and not for the individual TFs. This is due to the fact that individual binding site data for each TF within a family is not available. On the other hand, even though the TFs within a family may have unique functions and target genes, they may still share similar binding sites and may be involved in similar biological processes. Thus, applying the method at the gene family level will in many cases identify relevant interactions that can be further analysed experimentally.

From the above analysis it is clear that our model created an elaborate putative network is in which several previously known interactions were correctly predicted. In addition several novel interactions between key genes involved in the genetic control of cold acclimation in plants was suggested. These can now be directly addressed experimentally. In addition, since the model is general, it could in principle be used to study networks regulating any biological process in any biological systems. As far as cold stress is concerned it could pave the way for identification of useful molecular markers or relevant mutagenesis experiments in the development of cold tolerant commercial plant species.

## Conclusion

We here presented a novel approach to identify putative target genes for transcription factors where the binding sites are known. This approach utilizes upstream sequences, expression data and functional annotation to build large-scale regulatory networks. Such networks will be useful in studying regulatory activities in the cell at the molecular level. When we applied this model to the plant cold acclimation data, we were able to predict several new putative targets of known transcription factors. We also generated a large-scale genetic network related to cold acclimation in *Arabidopsis*. However, the approach presented here can of course easily be adapted to any species as long as the model requirements are fulfilled. Since the genomes of several organisms already have been sequenced and new genomes are continuously being added, the method presented here may serve as an additional tool to explore and validate important regulatory pathways and mechanisms in various biological systems.

## Methods

In overview, the method consists of a step-wise application of three constraints used to infer clusters of genes that are potentially regulated by the same combination of TFs. First, all genes in each cluster must contain in their upstream regions a sufficient number of occurrences of the same known motif or combination of motifs, for which there is a known TF-binding site. Second, the earliest recorded time-point of significant expression of each gene in the cluster must occur at the same time as the first recorded time-point of significant expression of the regulating TF-encoding gene, or at the immediately following time-point. Third, the expression profiles of the genes in the cluster must show a higher correlation than the expression profiles of randomly selected genes. Applying these three constraints leads to the formation of a grouping of the genes, based on which a regulatory network is derived by linking each known TF to the group(s) of co-expressed genes that it regulates.

### Data set and analysis

A publicly available Affymetrix microarray dataset comprising ~24,000 genes was downloaded from AtGenExpress. At AtGenExpress, 18 days old *Arabidopsis thaliana *plants of ecotype Columbia-0 were exposed to cold stress at 4°C. The material was harvested at 0.5 h, 1 h, 3 h, 6 h, 12 h and 24 h and transcript abundance was measured on the Affymetrix ATH1 chip. We downloaded the normalized data set and analyzed it using the GeneSpring™ 7.1 software (Silicon Genetics, Redwood City, CA, USA). Probe sets that met the following criteria were determined to be induced by cold and therefore selected for further analysis: a) those having an absolute call of "present" or "marginal" (*p *≤ 0.06) in at least half of the samples; b) determined to be up- or down-regulated by at least 2.5 folds at one or more time-points; and c) passed the ANOVA (*p *≤ 0.05) and the Benjamin and Hochberg False Discovery Rate multiple testing correction. Thus, out of ~24,000 probe sets, 1665 and 1830 probes passed all these criteria for up- and down-regulated genes, respectively. Of these, 134 genes code for DNA binding proteins (data not shown). Since most of these proteins were identified only recently, not much information is available about their preferred binding sites. Thus, information about known or putative binding sites (consensus sequence) of only 33 proteins could be collected (table 1). These proteins were grouped into 11 gene families and putative gene targets of these families were searched. For binding site analysis, the consensus sequence of the binding site was used for each family, if possible. In cases where a consensus could not be formed, all patterns were separately analysed. For CBFs, the pattern "CCGAC" was selected instead of "A/GCCGAC". This was done in order to not miss the lesser-known targets of CBFs. Thus 53% of the putative targets of CBFs identified by our approach contain the pattern "A/GCCGAC", while the remaining 47% of the targets contain "T/CCCGAC" in their 1 kb upstream promoters.

In *Arabidopsis*, functional *cis*-elements are normally located rather close to the RNA polymerase binding sites. A distance of ca 1000 bp 1 kb upstream region will therefore most often include all essential elements [35,51]. For this reason we choose to download all promoter upstream sequences ranging from position -1000 to -1 (the ATG translational start site was defined as position +1) of all the predicted *Arabidopsis thaliana *genes from the TAIR database [52,53]. Since *cis*-elements in 5'UTR have been shown to be involved in the regulation of e.g the *A1 *gene in *A. thaliana *[54], we also included the 5' UTRs in our analysis.

### Over-representation of binding motifs

For estimating the over-representation of the motifs within the 1 kb upstream region, *n*_*m *_(the number of occurrences of motif *m *upstream of a particular gene *n*) was compared with the average frequency of occurrence of *m *in all upstream sequences, *f*(*m*). The motif was selected if *n*_*m *_was found to be higher than the upper bound of the 95% confidence interval of *f*(*m*). For each motif pattern *m*, we calculated the total number of occurrences *M *in the upstream regions of the *N *genes in the whole genome. The average frequency of occurrence *f*(*m*) of *m *is then:

f(m)=MiN
 MathType@MTEF@5@5@+=feaafiart1ev1aaatCvAUfKttLearuWrP9MDH5MBPbIqV92AaeXatLxBI9gBaebbnrfifHhDYfgasaacH8akY=wiFfYdH8Gipec8Eeeu0xXdbba9frFj0=OqFfea0dXdd9vqai=hGuQ8kuc9pgc9s8qqaq=dirpe0xb9q8qiLsFr0=vr0=vr0dc8meaabaqaciaacaGaaeqabaqabeGadaaakeaacqWGMbGzcqGGOaakcqWGTbqBcqGGPaqkcqGH9aqpdaWcaaqaaiabd2eannaaBaaaleaacqWGPbqAaeqaaaGcbaGaemOta4eaaaaa@3605@

The 95% confidence interval (CI) can then be defined as:

(f(m)−z*σ2N) to (f(m)+z*σ2N)
 MathType@MTEF@5@5@+=feaafiart1ev1aaatCvAUfKttLearuWrP9MDH5MBPbIqV92AaeXatLxBI9gBaebbnrfifHhDYfgasaacH8akY=wiFfYdH8Gipec8Eeeu0xXdbba9frFj0=OqFfea0dXdd9vqai=hGuQ8kuc9pgc9s8qqaq=dirpe0xb9q8qiLsFr0=vr0=vr0dc8meaabaqaciaacaGaaeqabaqabeGadaaakeaadaqadaqaaiabdAgaMjabcIcaOiabd2gaTjabcMcaPiabgkHiTiabdQha6jabcQcaQmaalaaabaacciGae83Wdm3aaWbaaSqabeaacqaIYaGmaaaakeaadaGcaaqaaiabd6eaobqabaaaaaGaayjkaiaawMcaaiabbccaGGqaciab+rha0jab+9gaVjabbccaGmaabmaabaGaemOzayMaeiikaGIaemyBa0MaeiykaKIaey4kaSIaemOEaONaeiOkaOYaaSaaaeaacqWFdpWCdaahaaWcbeqaaiabikdaYaaaaOqaamaakaaabaGaemOta4eabeaaaaaacaGLOaGaayzkaaaaaa@4BDE@

where *σ*^2 ^is the standard deviation of *f*(*m*) and *z *is the *z*-value for the 95% CI. The upstream regions of all the genes were analyzed for motifs using the Patmatch tool and the binding site consensus sequences in table 1. For each motif *m*, we calculated *f*(*m*) using eq. 1, and the standard deviation *σ*^2 ^and the 95% CI using eq. 2. We let the number of occurrences of *M *upstream of a particular gene *n *be denoted by *n*_*M *_and considered motif *m *as over-represented for gene *n *if *n*_*M *_was larger than the upper threshold of the CI for the motif pattern.

### Time-order dependencies between TFs and target genes

Given a potential target gene *g*, the quantity *e*_*g*_(*t*) represents the expression level of *g *at time-point *t*. The "time of initiation of expression" of gene *g *is the first time-point at which *e*_*g*_(*t*) is greater than or equal to 1.1 folds of that of its control. Similarly, *e*_*tf*_(*t*) represents the expression level at time-point *t *of the transcription factor *tf *that potentially regulates *g*. In order to include *e*_*g*_(*t*) in the cluster of target genes of *tf*, the initiation of expression of *g *must occur at the same or the immediately following time-point to that of *tf*. For instances such as the WRKY family of TFs, where different TFs are known to recognize the same consensus sequence (table 1), all the TFs belonging to the same family were grouped together, and the time of initiation of expression of the TF family was calculated as the time interval between the earliest time point at which any of the TFs belonging to the family was expressed and the immediately proceeding time point at which the last TF was expressed. Thus for each TF family, a range of time-points was identified, between which all the TFs belonging to that family initiated to express.

### Expression coherence

Given a cluster of genes *S *containing a particular over-represented motif or motif combination in their upstream regions, and the gene expression data, the Pearson correlation coefficient *r *of each of the *p *= |*S*| * (|*S*| - 1)/2 pairs of genes can be calculated by:

r=∑i=1nxiyi−[∑i=1nxi∑i=1nyin][∑i=1nxi2−[∑i=1nxi]2n][∑i=1nyi2−[∑i=1nyi]2n]
 MathType@MTEF@5@5@+=feaafiart1ev1aaatCvAUfKttLearuWrP9MDH5MBPbIqV92AaeXatLxBI9gBaebbnrfifHhDYfgasaacH8akY=wiFfYdH8Gipec8Eeeu0xXdbba9frFj0=OqFfea0dXdd9vqai=hGuQ8kuc9pgc9s8qqaq=dirpe0xb9q8qiLsFr0=vr0=vr0dc8meaabaqaciaacaGaaeqabaqabeGadaaakeaacqWGYbGCcqGH9aqpdaWcaaqaamaaqahabaGaemiEaG3aaSbaaSqaaiabdMgaPbqabaGccqWG5bqEdaWgaaWcbaGaemyAaKgabeaakiabgkHiTmaadmaabaWaaSaaaeaadaaeWbqaaiabdIha4naaBaaaleaacqWGPbqAaeqaaaqaaiabdMgaPjabg2da9iabigdaXaqaaiabd6gaUbqdcqGHris5aOWaaabCaeaacqWG5bqEdaWgaaWcbaGaemyAaKgabeaaaeaacqWGPbqAcqGH9aqpcqaIXaqmaeaacqWGUbGBa0GaeyyeIuoaaOqaaiabd6gaUbaaaiaawUfacaGLDbaaaSqaaiabdMgaPjabg2da9iabigdaXaqaaiabd6gaUbqdcqGHris5aaGcbaWaaOaaaeaadaWadaqaamaaqahabaGaemiEaG3aa0baaSqaaiabdMgaPbqaaiabikdaYaaakiabgkHiTmaalaaabaWaamWaaeaadaaeWbqaaiabdIha4naaBaaaleaacqWGPbqAaeqaaaqaaiabdMgaPjabg2da9iabigdaXaqaaiabd6gaUbqdcqGHris5aaGccaGLBbGaayzxaaWaaWbaaSqabeaacqaIYaGmaaaakeaacqWGUbGBaaaaleaacqWGPbqAcqGH9aqpcqaIXaqmaeaacqWGUbGBa0GaeyyeIuoaaOGaay5waiaaw2faamaadmaabaWaaabCaeaacqWG5bqEdaqhaaWcbaGaemyAaKgabaGaeGOmaidaaOGaeyOeI0YaaSaaaeaadaWadaqaamaaqahabaGaemyEaK3aaSbaaSqaaiabdMgaPbqabaaabaGaemyAaKMaeyypa0JaeGymaedabaGaemOBa4ganiabggHiLdaakiaawUfacaGLDbaadaahaaWcbeqaaiabikdaYaaaaOqaaiabd6gaUbaaaSqaaiabdMgaPjabg2da9iabigdaXaqaaiabd6gaUbqdcqGHris5aaGccaGLBbGaayzxaaaaleqaaaaaaaa@8D74@

where *n *is the total number of gene expression experiments (time-points) available, and *x*_*i *_and *y*_*i *_are the expression values of genes *x *and *y *at time-point *i*. The expression coherence (EC) score *E *associated with the gene cluster *S *is defined as *p*/*P *where *p *is the number of gene pairs in *S *having *r *> *D*. For calculation of the threshold *D*, 100 genes were randomly selected from the set of ~24,000 genes and the Pearson correlation score was calculated between all possible gene pairs. The lowest value in the 75^th ^percentile of the obtained scores was considered as the value for *D*. The resultant *E *for a cluster *S *containing *N *genes is accepted if it is significantly greater than the lower threshold of the 95% confidence interval. The lower threshold for *E *was estimated by randomly selecting a set of *N *genes from the set of ~24,000 genes and calculating its EC score *e*. The average *e *obtained over 50 iterations was considered as the lower threshold.

## Authors' contributions

AC designed, developed and implemented the methodology. BO, OO, MB and AL gave suggestions during method development. OO and MB participated in the biological interpretation of results. BO coordinated the research. AC, MB, BO and OO drafted sections of the manuscript. All authors participated in editing and improving the manuscript.

## Supplementary Material

Additional file 1 Putative genetic network of the up-regulated genes. This data contains the list of up-regulated genes and their putative regulating factors predicted by this approach. Click here for file

Additional file 2 Promoter analysis of the up-regulated genes. This data shows the locations of motifs found in the promoter regions of the up-regulated genes. Click here for file

Additional file 3 Putative genetic network of the down-regulated genes. This data contains the list of down-regulated genes and their putative regulating factors predicted by this approach. Click here for file

Additional file 4 Promoter analysis of the down-regulated genes. This data shows the locations of motifs found in the promoter region of the down-regulated genes. Click here for file

Additional file 5 Functional annotations of the down-regulated genes. This data shows the over-represented functional annotations of the putative TF targets in the down-regulated genes. Click here for file

Additional file 6 Analysis of predicted CBF target genes. It shows the comparison analysis of the previously known down-stream targets of the CBF TFs to that predicted by this approach. Click here for file
