# Supplementary material for: Putative cold acclimation pathways in Arabidopsis thaliana identified by a combined analysis of mRNA co-expression patterns, promoter motifs and transcription factors
Source: BMC Genomics. 2007 Sep 2;8:304. doi: 10.1186/1471-2164-8-304 (PMC2001198; doi:10.1186/1471-2164-8-304)
Supplement: Additional file 3 — Putative genetic network of the down-regulated genes. This data contains the list of down-regulated genes and their putative regulating factors predicted by this approach. [file 1471-2164-8-304-S3.doc]

**Additional file 3**: Down regulated genes and their predicted regulators.

| **Target gene** | **Predicted Regulator** |
| --- | --- |
| At3g20680 | MYB,AP2(CBF) |
| At3g53100 | MYB,AP2(CBF) |
| At3g16120 | MYB,AP2(CBF) |
| At1g18485 | MYB,AP2(CBF) |
| At2g31150 | MYB,AP2(CBF) |
| At1g24020 | MYB,AP2(CBF) |
| At3g16250 | MYB,AP2(CBF) |
| At1g26590 | DOF,WRKY,AP2(CBF) |
| At5g35840 | DOF,WRKY,AP2(CBF) |
| At1g74600 | DOF,WRKY,AP2(CBF) |
| At4g12980 | DOF,WRKY,AP2(CBF) |
| At1g64330 | DOF,WRKY,AP2(CBF) |
| At2g46030 | DOF,WRKY,AP2(CBF) |
| At1g44920 | DOF,WRKY,AP2(CBF) |
| At4g23260 | DOF,WRKY,AP2(CBF) |
| At2g22250 | DOF,WRKY,AP2(CBF) |
| At1g75820 | DOF,WRKY,AP2(CBF) |
| At3g56000 | DOF,WRKY,AP2(CBF) |
| At4g30340 | DOF,WRKY,AP2(CBF) |
| At1g01620 | DOF,WRKY,AP2(CBF) |
| At4g30690 | DOF,WRKY,AP2(CBF) |
| At3g22970 | DOF,WRKY,AP2(CBF) |
| At2g46380 | DOF,WRKY,AP2(CBF) |
| At5g42130 | DOF,WRKY,AP2(CBF) |
| At4g00270 | DOF,AP2(CBF) |
| At2g45450 | DOF,AP2(CBF) |
| At4g11900 | DOF,AP2(CBF) |
| At2g47010 | DOF,AP2(CBF) |
| At1g21060 | DOF,AP2(CBF) |
| At4g13420 | DOF,AP2(CBF) |
| At3g62980 | DOF,AP2(CBF) |
| At3g16750 | DOF,AP2(CBF) |
| At1g45145 | DOF,AP2(CBF) |
| At4g27240 | DOF,AP2(CBF) |
| At2g01630 | DOF,AP2(CBF) |
| At4g21400 | DOF,AP2(CBF) |
| At1g12450 | DOF,AP2(CBF) |
| At1g58370 | DOF,AP2(CBF) |
| At4g12420 | DOF,AP2(CBF) |
| At5g01610 | DOF,AP2(CBF) |
| At3g25070 | DOF,AP2(CBF) |
| At1g18650 | DOF,AP2(CBF) |
| At4g13670 | DOF,AP2(CBF) |
| At1g17340 | DOF,AP2(CBF) |
| At5g56040 | DOF,AP2(CBF) |
| At3g12110 | DOF,AP2(CBF) |
| At3g24320 | DOF,AP2(CBF) |
| At3g02120 | DOF,AP2(CBF) |
| At3g01980 | DOF,AP2(CBF) |
| At2g42760 | DOF,AP2(CBF) |
| At3g14570 | DOF,AP2(CBF) |
| At3g25570 | DOF,AP2(CBF) |
| At5g44660 | DOF,AP2(CBF) |
| At1g72630 | DOF,AP2(CBF) |
| At1g18880 | DOF,AP2(CBF) |
| At1g62960 | DOF,AP2(CBF) |
| At5g50160 | DOF,AP2(CBF) |
| At1g23030 | DOF,AP2(CBF) |
| At5g07690 | DOF,AP2(CBF) |
| At3g09780 | DOF,AP2(CBF) |
| At4g28780 | DOF,AP2(CBF) |
| At4g31600 | DOF,AP2(CBF) |
| At4g02900 | DOF,AP2(CBF) |
| At2g38440 | DOF,AP2(CBF) |
| At4g37300 | DOF,AP2(CBF) |
| At3g21790 | DOF,AP2(CBF) |
| At4g34220 | DOF,AP2(CBF) |
| At3g62010 | DOF,AP2(CBF) |
| At5g11670 | DOF,AP2(CBF) |
| At4g32980 | DOF,AP2(CBF) |
| At4g33960 | DOF,AP2(CBF) |
| At2g16660 | DOF,AP2(CBF) |
| At5g59130 | DOF,AP2(CBF) |
| At5g44410 | DOF,AP2(CBF) |
| At4g34138 | DOF,AP2(CBF) |
| At2g13690 | DOF,AP2(CBF) |
| At1g27120 | DOF,AP2(CBF) |
| At5g53940 | DOF,AP2(CBF) |
| At1g62200 | DOF,AP2(CBF) |
| At2g03310 | DOF,AP2(CBF) |
| At5g03440 | DOF,AP2(CBF) |
| At4g19420 | DOF,AP2(CBF) |
| At2g20650 | DOF,AP2(CBF) |
| At3g11280 | DOF,AP2(CBF) |
| At3g53950 | DOF,AP2(CBF) |
| At4g19410 | DOF,AP2(CBF) |
| At4g34020 | DOF,AP2(CBF) |
| At5g47520 | DOF,AP2(CBF) |
| At4g13410 | BHLH |
| At5g42800 | BHLH |
| At5g16940 | BHLH |
| At5g05960 | BHLH |
| At5g54520 | BHLH |
| At1g70270 | BHLH |
| At4g39940 | BHLH |
| At3g25690 | BHLH |
| At4g26160 | BHLH |
| At4g19430 | BHLH |
| At2g34150 | BHLH |
| At5g22860 | BHLH |
| At2g20610 | BHLH |
| At1g60690 | BHLH |
| At4g38225 | BHLH |
| At1g70250 | BHLH |
| At1g15960 | BHLH |
| At2g43100 | BHLH |
| At5g56590 | BHLH |
| At4g38670 | BHLH |
| At4g15440 | BHLH |
| At3g07140 | BHLH |
| At4g24760 | BHLH |
| At5g45670 | BHLH |
| At4g10770 | BHLH |
| At1g15180 | BHLH |
| At4g14600 | BHLH |
| At3g63460 | BHLH |
| At1g75130 | BHLH |
| At4g21650 | BHLH |
| At5g56860 | BHLH |
| At1g09740 | BHLH |
| At5g66620 | BHLH |
| At5g25090 | BHLH |
| At5g07130 | BHLH |
| At1g20190 | BHLH |
| At1g74670 | BHLH |
| At5g64900 | BHLH |
| At1g67750 | BHLH |
| At4g24350 | BHLH |
| At1g26220 | BHLH |
| At2g42950 | BHLH |
| At3g59180 | BHLH |
| At1g75220 | BHLH |
| At3g52290 | BHLH |
| At4g28680 | BHLH |
| At5g35740 | BHLH |
| At1g12220 | BHLH |
| At4g09620 | BHLH |
| At2g23290 | BHLH |
| At1g48480 | BHLH |
| At5g58140 | BHLH |
| At1g23480 | BHLH |
| At3g59490 | BHLH |
| At1g70160 | BHLH |
| At1g12280 | BHLH |
| At1g51680 | BHLH |
| At1g64400 | BHLH |
| At3g56750 | BHLH |
| At4g26520 | BHLH |
| At1g11440 | BHLH |
| At2g03550 | BHLH |
| At4g24780 | BHLH |
| At1g70580 | BHLH |
| At1g78680 | BHLH |
| At5g03140 | BHLH |
| At4g13050 | BHLH |
| At4g22340 | BHLH |
| At3g11110 | BHLH |
| At4g12390 | BHLH |
| At2g34310 | BHLH |
| At1g74090 | BHLH |
| At3g26450 | BHLH |
| At1g72300 | BHLH |
| At2g29340 | BHLH |
| At1g51360 | BHLH |
| At4g22200 | BHLH |
| At1g29500 | BHLH |
| At1g71880 | BHLH |
| At2g07680 | BHLH |
| At1g68130 | BHLH |
| At3g10150 | BHLH |
| At2g20500 | BHLH |
| At4g26555 | BHLH |
| At4g00750 | BHLH |
| At1g51430 | BHLH |
| At1g10240 | BHLH |
| At5g13770 | BHLH |
| At2g24160 | BHLH |
| At4g16980 | BHLH |
| At3g54220 | BHLH |
| At2g47630 | BHLH |
| At3g12610 | BHLH |
| At1g71180 | BHLH |
| At1g62250 | BHLH |
| At1g15260 | BHLH |
| At4g26850 | BHLH |
| At5g38980 | BHLH |
| At5g02830 | BHLH |
| At5g49100 | BHLH |
| At1g14120 | BHLH |
| At3g48200 | BHLH |
| At2g31110 | BHLH |
| At5g42680 | BHLH |
| At3g18800 | BHLH |
| At1g69530 | BHLH |
| At1g65230 | BHLH |
| At1g53450 | BHLH |
| At5g10960 | BHLH |
| At5g42750 | BHLH |
| At1g56700 | BHLH |
| At1g26920 | BHLH |
| At2g04940 | BHLH |
| At3g02170 | BHLH |
| At5g15230 | BHLH |
| At4g17080 | BHLH |
| At3g48460 | BHLH |
| At3g23410 | BHLH |
| At5g02560 | BHLH |
| At3g23310 | BHLH |
| At5g01015 | BHLH |
| At1g55910 | BHLH |
| At2g22740 | BHLH |
| At5g55120 | BHLH |
| At1g19000 | BHLH |
| At2g15580 | BHLH |
| At3g17330 | BHLH |
| At4g19860 | BHLH |
| At2g23130 | BHLH |
| At2g20830 | BHLH |
| At1g70210 | BHLH |
| At4g30993 | BHLH |
| At2g24150 | BHLH |
| At1g75960 | BHLH |
| At1g70820 | BHLH |
| At1g74530 | BHLH |
| At4g18020 | BHLH |
| At4g32570 | BHLH |
| At4g29220 | BHLH |
| At4g12830 | BHLH |
| At4g25720 | BHLH |
| At1g14360 | BHLH |
| At4g24670 | BHLH |
| At3g26590 | BHLH |
| At1g60600 | BHLH |
| At3g23880 | BHLH |
| At1g10960 | BHLH |
| At1g15290 | BHLH |
| At2g31670 | BHLH |
| At4g29200 | DOF,MYB |
| At5g03350 | DOF,MYB |
| At1g29950 | DOF,MYB |
| At5g25120 | DOF,MYB |
| At3g22540 | DOF,MYB |
| At5g65040 | DOF,MYB |
| At1g14200 | DOF,MYB |
| At4g01460 | DOF,MYB |
| At1g13090 | DOF,MYB |
| At1g68585 | DOF,MYB |
| At5g47500 | DOF,MYB |
| At1g24440 | DOF,MYB |
| At2g17970 | DOF,MYB |
| At1g62660 | DOF,MYB |
| At4g19670 | DOF,MYB |
| At4g27710 | DOF,MYB |
| At3g24770 | DOF,MYB |
| At3g21750 | DOF,MYB |
| At1g70550 | DOF,MYB |
| At5g37680 | DOF,MYB |
| At4g03070 | DOF,MYB |
| At1g14340 | DOF,MYB |
| At4g10060 | DOF,MYB |
| At2g21530 | DOF,MYB |
| At5g63780 | DOF,MYB |
| At2g31790 | DOF,MYB |
| At2g22330 | DOF,MYB |
| At1g26690 | DOF,WRKY,BHLH,AP2(CBF),HSF |
| At3g51750 | DOF,WRKY,BHLH,AP2(CBF),HSF |
| At1g52260 | DOF,WRKY,BHLH,AP2(CBF),HSF |
| At4g28640 | DOF,WRKY,BHLH,AP2(CBF),HSF |
| At1g03090 | DOF,WRKY,BHLH,AP2(CBF),HSF |
| At2g42300 | DOF,WRKY,BHLH,AP2(CBF),HSF |
| At1g52250 | DOF,WRKY,BHLH,AP2(CBF),HSF |
| At4g37550 | DOF,WRKY,BHLH,AP2(CBF),HSF |
| At2g18670 | DOF,WRKY,BHLH,AP2(CBF),HSF |
| At5g14430 | DOF,WRKY,BHLH,AP2(CBF),HSF |
| At1g06550 | DOF,WRKY,BHLH,AP2(CBF),HSF |
| At3g48720 | DOF,WRKY,BHLH,AP2(CBF),HSF |
| At2g44210 | DOF,WRKY,BHLH,AP2(CBF),HSF |
| At3g60910 | DOF,WRKY,BHLH,AP2(CBF),HSF |
| At2g38570 | AP2(CBF) |
| At1g11630 | AP2(CBF) |
| At2g25570 | AP2(CBF) |
| At3g13030 | AP2(CBF) |
| At1g05590 | AP2(CBF) |
| At2g02710 | AP2(CBF) |
| At3g56270 | AP2(CBF) |
| At5g37930 | AP2(CBF) |
| At2g07718 | AP2(CBF) |
| At5g05800 | AP2(CBF) |
| At1g34630 | AP2(CBF) |
| At1g20160 | AP2(CBF) |
| At2g30920 | AP2(CBF) |
| At2g21830 | AP2(CBF) |
| At4g14010 | AP2(CBF) |
| At1g66540 | AP2(CBF) |
| At2g45700 | AP2(CBF) |
| At3g52070 | AP2(CBF) |
| At4g14610 | AP2(CBF) |
| At2g29310 | AP2(CBF) |
| At1g08250 | AP2(CBF) |
| At2g14750 | AP2(CBF) |
| At3g12950 | AP2(CBF) |
| At5g52550 | AP2(CBF) |
| At2g17640 | AP2(CBF) |
| At5g20320 | AP2(CBF) |
| At5g67580 | AP2(CBF) |
| At1g19960 | AP2(CBF) |
| At2g38300 | AP2(CBF) |
| At3g56040 | AP2(CBF) |
| At3g11620 | AP2(CBF) |
| At3g51420 | AP2(CBF) |
| At5g08330 | AP2(CBF) |
| At1g05860 | AP2(CBF) |
| At4g38690 | AP2(CBF) |
| At3g05180 | AP2(CBF) |
| At5g27950 | AP2(CBF) |
| At1g24100 | AP2(CBF) |
| At1g31690 | AP2(CBF) |
| At5g58480 | AP2(CBF) |
| At1g01430 | AP2(CBF) |
| At1g21600 | AP2(CBF) |
| At5g60800 | AP2(CBF) |
| At5g14450 | AP2(CBF) |
| At1g69160 | AP2(CBF) |
| At1g06460 | AP2(CBF) |
| At4g17350 | AP2(CBF) |
| At1g03410 | AP2(CBF) |
| At1g09795 | AP2(CBF) |
| At3g23570 | AP2(CBF) |
| At3g49050 | AP2(CBF) |
| At3g17040 | AP2(CBF) |
| At5g26820 | AP2(CBF) |
| At1g14280 | AP2(CBF) |
| At3g13780 | BHLH,HSF |
| At5g15780 | BHLH,HSF |
| At1g54100 | BHLH,HSF |
| At5g05660 | BHLH,HSF |
| At2g18770 | BHLH,HSF |
| At1g76930 | BHLH,HSF |
| At4g16710 | BHLH,HSF |
| At1g47400 | BHLH,HSF |
| At3g04290 | BHLH,HSF |
| At1g64670 | BHLH,HSF |
| At3g07580 | BHLH,HSF |
| At4g17070 | BHLH,HSF |
| At4g33580 | BHLH,HSF |
| At5g40500 | BHLH,HSF |
| At2g19880 | BHLH,HSF |
| At1g09415 | BHLH,HSF |
| At5g46790 | BHLH,HSF |
| At1g13990 | BHLH,HSF |
| At5g67390 | BHLH,HSF |
| At1g03870 | BHLH,HSF |
| At4g33750 | BHLH,HSF |
| At5g64880 | BHLH,HSF |
| At5g02480 | BHLH,HSF |
| At1g55250 | BHLH,HSF |
| At2g42900 | BHLH,HSF |
| At4g23690 | BHLH,HSF |
| At5g06870 | BHLH,HSF |
| At4g23300 | BHLH,HSF |
| At1g01120 | BHLH,HSF |
| At1g78550 | BHLH,HSF |
| At2g46220 | BHLH,HSF |
| At4g25830 | BHLH,HSF |
| At3g07860 | WRKY,BHLH |
| At3g26720 | WRKY,BHLH |
| At1g33390 | WRKY,BHLH |
| At3g60620 | WRKY,BHLH |
| At3g56640 | WRKY,BHLH |
| At4g21990 | WRKY,BHLH |
| At5g55480 | WRKY,BHLH |
| At1g79460 | WRKY,BHLH |
| At1g56720 | WRKY,BHLH |
| At1g78670 | WRKY,BHLH |
| At2g01450 | WRKY,BHLH |
| At1g05670 | WRKY,BHLH |
| At1g31820 | WRKY,BHLH |
| At4g31050 | WRKY,BHLH |
| At3g24040 | WRKY,BHLH |
| At3g26230 | WRKY,BHLH |
| At5g16800 | WRKY,BHLH |
| At5g46270 | WRKY,BHLH |
| At1g70660 | WRKY,BHLH |
| At1g65860 | WRKY,BHLH |
| At3g23805 | WRKY,BHLH |
| At1g29600 | WRKY,BHLH |
| At3g56060 | WRKY,BHLH |
| At1g77870 | WRKY,BHLH |
| At1g54730 | WRKY,BHLH |
| At2g40840 | WRKY,BHLH |
| At1g77020 | DOF,HSF |
| At1g66350 | DOF,HSF |
| At2g05540 | DOF,HSF |
| At1g72830 | DOF,HSF |
| At5g16520 | DOF,HSF |
| At1g05470 | DOF,HSF |
| At1g02300 | DOF,HSF |
| At2g28630 | DOF,HSF |
| At1g02850 | DOF,HSF |
| At1g21250 | DOF,HSF |
| At1g28580 | DOF,HSF |
| At5g52280 | DOF,HSF |
| At3g57630 | DOF,HSF |
| At2g31040 | DOF,HSF |
| At1g62560 | DOF,HSF |
| At3g28140 | DOF,HSF |
| At1g55330 | DOF,HSF |
| At3g60130 | DOF,HSF |
| At1g74710 | DOF,HSF |
| At5g14120 | DOF,HSF |
| At4g04840 | DOF,HSF |
| At5g42890 | DOF,HSF |
| At4g19660 | DOF,HSF |
| At5g44060 | DOF,HSF |
| At3g08000 | DOF,HSF |
| At3g45210 | DOF,HSF |
| At3g09920 | DOF,HSF |
| At3g12700 | DOF,HSF |
| At3g43670 | DOF,HSF |
| At2g17550 | DOF,HSF |
| At5g59670 | DOF,HSF |
| At3g23760 | DOF,HSF |
| At4g20270 | DOF,HSF |
| At3g61210 | DOF,HSF |
| At2g18440 | DOF,HSF |
| At3g06330 | DOF,HSF |
| At1g14440 | DOF,HSF |
| At3g45240 | DOF,HSF |
| At4g16447 | DOF,HSF |
| At2g37460 | DOF,HSF |
| At2g45560 | DOF,HSF |
| At4g35270 | DOF,HSF |
| At3g59780 | DOF,HSF |
| At2g40670 | DOF,HSF |
| At3g54810 | DOF,HSF |
| At2g34660 | DOF,HSF |
| At5g62730 | DOF,HSF |
| At1g16750 | DOF,HSF |
| At5g40380 | DOF,HSF |
| At1g50280 | DOF,HSF |
| At5g55620 | DOF,HSF |
| At5g18650 | DOF,HSF |
| At5g44680 | DOF,HSF |
| At2g24762 | DOF,HSF |
| At4g20930 | DOF,HSF |
| At4g19530 | DOF,HSF |
| At4g21810 | DOF,HSF |
| At2g46450 | DOF,HSF |
| At5g15350 | DOF,HSF |
| At1g05460 | DOF,BHLH |
| At1g78970 | DOF,BHLH |
| At1g26770 | DOF,BHLH |
| At2g22770 | DOF,BHLH |
| At2g15280 | DOF,BHLH |
| At2g20750 | DOF,BHLH |
| At5g12900 | DOF,BHLH |
| At1g79410 | DOF,BHLH |
| At2g42960 | DOF,BHLH |
| At1g17610 | DOF,BHLH |
| At3g28100 | DOF,BHLH |
| At4g12030 | DOF,BHLH |
| At3g52900 | DOF,BHLH |
| At2g41310 | DOF,BHLH |
| At2g26870 | DOF,BHLH |
| At5g16190 | DOF,BHLH |
| At2g17530 | DOF,BHLH |
| At4g17245 | DOF,BHLH |
| At2g15040 | DOF,BHLH |
| At2g42370 | DOF,BHLH |
| At2g23610 | DOF,BHLH |
| At1g77270 | DOF,BHLH |
| At1g47340 | DOF,BHLH |
| At4g14020 | DOF,BHLH |
| At4g09520 | DOF,BHLH |
| At3g13750 | DOF,BHLH |
| At2g47450 | DOF,BHLH |
| At1g12320 | DOF,BHLH |
| At2g30890 | DOF,BHLH |
| At2g22090 | DOF,BHLH |
| At1g01070 | DOF,BHLH |
| At1g49470 | DOF,BHLH |
| At5g46690 | DOF,BHLH |
| At4g02420 | DOF,BHLH |
| At1g11410 | DOF,BHLH |
| At5g06670 | DOF,BHLH |
| At4g17790 | DOF,BHLH |
| At4g08160 | DOF,BHLH |
| At5g52120 | DOF,BHLH |
| At4g00880 | DOF,BHLH |
| At4g27230 | DOF,BHLH |
| At5g48500 | DOF,BHLH |
| At3g06850 | DOF,BHLH |
| At2g41140 | DOF,BHLH |
| At3g46130 | DOF,BHLH |
| At2g07140 | DOF,BHLH |
| At1g69780 | DOF,BHLH |
| At1g55920 | DOF,BHLH |
| At5g65760 | DOF,BHLH |
| At4g17030 | DOF,BHLH |
| At5g15830 | DOF,BHLH |
| At4g16370 | DOF,BHLH |
| At3g27420 | DOF,BHLH |
| At5g64380 | DOF,BHLH |
| At5g57900 | DOF,BHLH |
| At3g02690 | DOF,BHLH |
| At2g27820 | DOF,BHLH |
| At4g29210 | DOF,BHLH |
| At1g77060 | DOF,BHLH |
| At3g61950 | DOF,BHLH |
| At5g40450 | DOF,BHLH |
| At1g20010 | DOF,BHLH |
| At5g65380 | DOF,BHLH |
| At5g23680 | DOF,BHLH |
| At2g16280 | DOF,BHLH |
| At2g30460 | DOF,BHLH |
| At4g02440 | DOF,BHLH |
| At1g25230 | DOF,BHLH |
| At2g38970 | DOF,BHLH |
| At1g14345 | DOF,BHLH |
| At3g26932 | DOF,BHLH |
| At4g34760 | DOF,BHLH |
| At2g18910 | DOF,BHLH |
| At3g13000 | DOF,BHLH |
| At3g11230 | DOF,BHLH |
| At2g46225 | DOF,BHLH |
| At1g48280 | DOF,BHLH |
| At5g35490 | DOF,BHLH |
| At5g12890 | DOF,BHLH |
| At3g14240 | DOF,BHLH |
| At4g13830 | DOF,BHLH |
| At5g53020 | DOF,BHLH |
| At1g69935 | DOF,BHLH |
| At5g59780 | DOF,BHLH |
| At5g64860 | DOF,BHLH |
| At3g45310 | DOF,BHLH |
| At2g32690 | DOF,BHLH |
| At4g21620 | DOF,BHLH |
| At3g26890 | DOF,BHLH |
| At2g42790 | DOF,BHLH |
| At1g70230 | DOF,BHLH |
| At2g01620 | DOF,BHLH |
| At5g23660 | DOF,BHLH |
| At2g19310 | DOF,BHLH |
| At4g30530 | DOF,BHLH |
| At5g08370 | DOF,BHLH |
| At5g41210 | DOF,BHLH |
| At4g03210 | DOF,BHLH |
| At1g25450 | DOF,BHLH |
| At4g02050 | DOF,BHLH |
| At2g14170 | DOF,BHLH |
| At3g47560 | DOF,BHLH |
| At2g47910 | DOF,BHLH |
| At5g08350 | DOF,BHLH |
| At2g22990 | DOF,BHLH |
| At5g54630 | DOF,BHLH |
| At2g20725 | DOF,BHLH |
| At3g13062 | DOF,BHLH |
| At5g05690 | DOF,BHLH |
| At3g18050 | DOF,BHLH |
| At4g01700 | WRKY,AP2(CBF),HSF |
| At4g17250 | WRKY,AP2(CBF),HSF |
| At3g26220 | WRKY,AP2(CBF),HSF |
| At3g26300 | WRKY,AP2(CBF),HSF |
| At3g21350 | WRKY,AP2(CBF),HSF |
| At2g37680 | WRKY,AP2(CBF),HSF |
| At1g01820 | WRKY,AP2(CBF),HSF |
| At4g13020 | WRKY,AP2(CBF),HSF |
| At1g77420 | WRKY,AP2(CBF),HSF |
| At2g07711 | WRKY,AP2(CBF),HSF |
| At5g20580 | WRKY,AP2(CBF),HSF |
| At1g27150 | WRKY,AP2(CBF),HSF |
| At3g29240 | WRKY,AP2(CBF),HSF |
| At1g08230 | WRKY,AP2(CBF),HSF |
| At3g07560 | WRKY,AP2(CBF),HSF |
| At1g65070 | WRKY,AP2(CBF),HSF |
| At1g15000 | DOF,BHLH, |
| At5g09460 | DOF,BHLH, |
| At2g39560 | DOF,BHLH, |
| At5g52220 | DOF,BHLH, |
| At1g17100 | DOF,BHLH, |
| At5g09820 | DOF,BHLH, |
| At5g20935 | DOF,BHLH, |
| At3g30720 | BZIP,HSF |
| At5g18850 | BZIP,HSF |
| At3g56330 | BZIP,HSF |
| At1g01490 | BZIP,HSF |
| At3g16690 | BZIP,HSF |
| At3g52370 | BHLH |
| At5g17400 | BHLH |
| At3g48490 | BHLH |
| At3g15680 | BHLH |
| At5g60230 | BHLH |
| At3g47540 | BHLH |
| At1g22160 | BHLH |
| At4g31115 | BHLH |
| At4g26790 | BHLH |
| At1g72970 | BHLH |
| At1g49930 | BHLH |
| At1g77670 | BHLH |
| At3g60690 | BHLH |
| At5g51970 | BHLH |
| At1g62950 | BHLH |
| At5g61340 | BHLH |
| At3g48690 | BHLH |
| At1g48320 | BHLH |
| At1g15410 | BHLH |
| At4g37560 | BHLH |
| At3g21870 | BHLH |
| At4g39510 | BHLH |
| At2g19780 | BHLH |
| At4g27600 | BHLH |
| At2g01260 | BHLH |
| At5g21100 | BHLH |
| At1g04350 | BHLH |
| At5g12310 | BHLH |
| At5g47610 | BHLH |
| At5g57800 | BHLH |
| At5g01920 | BHLH |
| At4g01450 | BHLH |
| At1g29720 | BHLH |
| At1g64200 | BHLH |
| At3g50750 | BHLH |
| At3g23200 | BHLH |
| At1g11580 | BHLH |
| At4g15550 | BHLH |
| At5g53370 | BHLH |
| At5g13400 | BHLh |
| At3g56140 | BHLH |
| At1g19650 | BHLH |
| At5g13640 | BHLH |
| At5g52420 | BHLH |
| At1g07350 | BHLH |
| At2g40020 | BHLH |
| At4g17340 | BHLH |
| At3g18750 | BHLH |
| At2g32010 | BHLH |
| At4g19820 | BHLH |
| At2g42690 | BHLH |
| At2g21970 | BHLH |
| At3g51950 | BHLH |
| At1g28560 | BHLH |
| At1g27360 | BHLH |
| At5g67230 | BHLH |
| At4g38950 | BHLH |
| At3g28040 | BHLH |
| At5g45410 | BHLH |
| At1g33811 | BHLH |
| At5g52780 | BHLH |
| At4g37330 | BHLH |
| At1g70000 | BHLH |
| At1g72900 | WRKY,HSF |
| At1g40150 | WRKY,HSF |
| At1g09160 | WRKY,HSF |
| At5g44070 | WRKY,HSF |
| At3g62330 | WRKY,HSF |
| At1g36380 | WRKY,HSF |
| At3g28200 | WRKY,HSF |
| At2g25420 | WRKY,HSF |
| At3g46940 | WRKY,HSF |
| At5g14410 | WRKY,HSF |
| At1g26580 | WRKY,HSF |
| At3g04210 | WRKY,HSF |
| At5g21070 | WRKY,HSF |
| At5g54610 | WRKY,HSF |
| At4g26760 | WRKY,HSF |
| At1g50730 | WRKY,HSF |
| At4g08690 | WRKY,HSF |
| At1g24240 | WRKY,HSF |
| At3g59400 | WRKY,HSF |
| At1g52190 | WRKY,HSF |
| At5g64410 | WRKY,HSF |
| At3g20120 | WRKY,HSF |
| At2g21050 | DOF,BHLH,HSF |
| At2g34810 | DOF,BHLH,HSF |
| At2g38760 | DOF,BHLH,HSF |
| At5g40920 | DOF,BHLH,HSF |
| At1g03030 | DOF,BHLH,HSF |
| At1g69770 | DOF,BHLH,HSF |
| At4g02610 | DOF,BHLH,HSF |
| At5g13760 | DOF,BHLH,HSF |
| At2g30540 | DOF,BHLH,HSF |
| At2g38090 | DOF,BHLH,HSF |
| At4g04610 | DOF,BHLH,HSF |
| At1g16090 | DOF,BHLH,HSF |
| At1g71340 | DOF,BHLH,HSF |
| At5g54380 | DOF,BHLH,HSF |
| At1g17745 | DOF,BHLH,HSF |
| At1g72680 | DOF,BHLH,HSF |
| At1g43650 | DOF,BHLH,HSF |
| At3g14920 | DOF,BHLH,HSF |
| At1g03457 | DOF,BHLH,HSF |
| At1g58080 | DOF,BHLH,HSF |
| At3g16570 | DOF,BHLH,HSF |
| At1g11350 | DOF,BHLH,HSF |
| At5g24520 | DOF,BHLH,HSF |
| At1g65660 | DOF,BHLH,HSF |
| At3g26740 | DOF,BHLH,HSF |
| At5g04470 | DOF,BHLH,HSF |
| At4g12690 | DOF,BHLH,HSF |
| At5g39790 | DOF,BHLH,HSF |
| At3g06130 | DOF,BHLH,HSF |
| At5g24430 | DOF,BHLH,HSF |
| At3g58120 | DOF,BHLH,HSF |
| At1g64640 | DOF,BHLH,HSF |
| At2g30520 | DOF,BHLH,HSF |
| At3g26280 | DOF,BHLH,HSF |
| At2g32720 | DOF,BHLH,HSF |
| At3g05910 | DOF,BHLH,HSF |
| At3g50070 | DOF,BHLH,HSF |
| At2g38310 | DOF,BHLH,HSF |
| At2g41090 | DOF,BHLH,HSF |
| At5g34940 | DOF,BHLH,HSF |
| At2g23180 | DOF,BHLH,HSF |
| At5g48490 | DOF,BHLH,HSF |
| At1g65900 | WRKY,BHLH,HSF |
| At4g20820 | WRKY,BHLH,HSF |
| At4g35420 | WRKY,BHLH,HSF |
| At3g61550 | WRKY,BHLH,HSF |
| At1g58180 | WRKY,BHLH,HSF |
| At1g28570 | WRKY,BHLH,HSF |
| At2g39440 | WRKY,BHLH,HSF |
| At4g26140 | WRKY,BHLH,HSF |
| At5g08210 | WRKY,BHLH,HSF |
| At4g26070 | WRKY,BHLH,HSF |
| At4g18910 | WRKY,BHLH,HSF |
| At4g33540 | WRKY,BHLH,HSF |
| At1g01770 | WRKY,BHLH,HSF |
| At2g48010 | WRKY,BHLH,HSF |
| At5g25630 | WRKY,BHLH,HSF |
| At4g38680 | WRKY,BHLH,HSF |
| At5g50180 | WRKY,BHLH,HSF |
| At3g04720 | WRKY,BHLH,HSF |
| At4g38860 | WRKY,BHLH,HSF |
| At1g70520 | WRKY,BHLH,HSF |
| At4g33490 | WRKY,BHLH,HSF |
| At1g70620 | WRKY,BHLH,HSF |
| At1g80180 | WRKY,BHLH,HSF |
| At4g38840 | WRKY,BHLH,HSF |
| At2g33330 | DOF,WRKY |
| At3g46580 | DOF,WRKY |
| At1g14330 | DOF,WRKY |
| At1g05710 | DOF,WRKY |
| At2g39400 | DOF,WRKY |
| At4g04740 | DOF,WRKY |
| At2g45340 | DOF,WRKY |
| At1g65620 | DOF,WRKY |
| At1g75840 | DOF,WRKY |
| At3g58975 | DOF,WRKY |
| At1g68060 | DOF,WRKY |
| At1g53730 | DOF,WRKY |
| At5g66490 | DOF,WRKY |
| At5g24165 | DOF,WRKY |
| At3g28920 | DOF,WRKY |
| At5g04020 | DOF,WRKY |
| At3g15095 | DOF,WRKY |
| At2g28410 | DOF,WRKY |
| At1g59700 | DOF,WRKY |
| At5g49980 | DOF,WRKY |
| At4g03110 | DOF,WRKY |
| At5g40020 | DOF,WRKY |
| At3g16230 | DOF,WRKY |
| At5g38530 | DOF,WRKY |
| At3g47640 | DOF,WRKY |
| At5g21170 | DOF,WRKY |
| At1g19835 | DOF,WRKY |
| At4g17970 | DOF,WRKY |
| At1g17360 | DOF,WRKY |
| At3g46900 | DOF,WRKY |
| At1g63260 | DOF,WRKY |
| At5g52870 | DOF |
| At5g24800 | DOF |
| At1g66890 | DOF |
| At5g58340 | DOF |
| At1g77460 | DOF |
| At1g15490 | DOF |
| At5g15210 | DOF |
| At1g60970 | DOF |
| At2g04570 | DOF |
| At3g29030 | DOF |
| At4g14750 | DOF |
| At3g45160 | DOF |
| At1g28100 | DOF |
| At5g04230 | DOF |
| At3g57800 | DOF |
| At1g64580 | DOF |
| At5g40550 | DOF |
| At1g09440 | DOF |
| At2g01830 | DOF |
| At5g51560 | DOF |
| At5g65640 | DOF |
| At4g26090 | DOF |
| At2g46650 | DOF |
| At2g45150 | DOF |
| At1g77920 | DOF |
| At5g24890 | DOF |
| At5g22940 | DOF |
| At4g09030 | DOF |
| At1g54390 | DOF |
| At2g46710 | DOF |
| At3g50630 | DOF |
| At3g11750 | DOF |
| At1g60800 | DOF |
| At5g46150 | DOF |
| At1g61350 | DOF |
| At4g04630 | DOF |
| At3g59300 | DOF |
| At4g08290 | DOF |
| At1g28110 | DOF |
| At5g65410 | DOF |
| At3g22550 | DOF |
| At5g23750 | DOF |
| At2g15050 | DOF |
| At3g60350 | DOF |
| At3g02910 | DOF |
| At3g01690 | DOF |
| At2g40435 | DOF |
| At2g31260 | DOF |
| At2g16270 | DOF |
| At1g36160 | DOF |
| At1g32540 | DOF |
| At5g10650 | DOF |
| At4g32710 | DOF |
| At3g26800 | DOF |
| At2g43530 | DOF |
| At4g21380 | DOF |
| At2g37130 | DOF |
| At3g23160 | DOF |
| At5g48640 | DOF |
| At1g27520 | DOF |
| At1g34210 | DOF |
| At2g37380 | DOF |
| At2g15680 | DOF |
| At1g29240 | DOF |
| At1g66970 | DOF |
| At3g49710 | DOF |
| At3g57070 | DOF |
| At3g28050 | DOF |
| At3g57830 | DOF |
| At1g66080 | DOF |
| At5g46830 | DOF |
| At1g12400 | DOF |
| At5g40150 | DOF |
| At1g13750 | DOF |
| At1g62030 | DOF |
| At3g45140 | DOF |
| At2g33480 | DOF |
| At2g03980 | DOF |
| At5g51770 | DOF |
| At1g16490 | DOF |
| At1g20090 | DOF |
| At1g07640 | DOF |
| At1g49740 | DOF |
| At5g64340 | DOF |
| At3g53190 | DOF |
| At1g21440 | DOF |
| At1g29260 | DOF |
| At5g08380 | DOF |
| At1g49980 | DOF |
| At3g22240 | DOF |
| At1g14920 | DOF |
| At5g19630 | DOF |
| At3g28910 | DOF |
| At2g26640 | DOF |
| At2g26980 | DOF |
| At5g35670 | DOF |
| At5g62980 | DOF |
| At1g19510 | DOF |
| At3g56950 | DOF |
| At2g22660 | DOF |
| At1g64720 | DOF |
| At1g27690 | DOF |
| At4g34630 | DOF |
| At1g25390 | DOF |
| At1g18620 | DOF |
| At1g60870 | DOF |
| At3g19800 | DOF |
| At1g78170 | DOF |
| At3g22210 | DOF |
| At4g22790 | DOF |
| At1g10200 | DOF |
| At4g15920 | DOF |
| At4g14930 | DOF |
| At1g67080 | DOF |
| At1g80690 | DOF |
| At5g35790 | DOF |
| At4g11660 | DOF |
| At4g37180 | DOF |
| At4g17870 | DOF |
| At4g18930 | DOF |
| At4g04955 | DOF |
| At4g36540 | DOF |
| At1g28400 | DOF |
| At3g11090 | DOF |
| At5g43270 | DOF |
| At3g23730 | DOF |
| At3g50500 | DOF |
| At1g56670 | DOF |
| At1g63800 | DOF |
| At3g05490 | DOF |
| At4g39900 | DOF |
| At3g60220 | DOF |
| At4g38060 | DOF |
| At3g11930 | DOF |
| At5g22850 | DOF |
| At1g54820 | DOF |
| At1g47740 | DOF |
| At4g01330 | DOF |
| At1g69910 | DOF,WRKY,BHLH |
| At3g20100 | DOF,WRKY,BHLH |
| At5g02890 | DOF,WRKY,BHLH |
| At2g39310 | DOF,WRKY,BHLH |
| At1g11330 | DOF,WRKY,BHLH |
| At5g04880 | DOF,WRKY,BHLH |
| At1g26210 | DOF,WRKY,BHLH |
| At3g45430 | DOF,WRKY,BHLH |
| At1g68220 | DOF,WRKY,BHLH |
| At2g21210 | DOF,WRKY,BHLH |
| At1g09250 | DOF,WRKY,BHLH |
| At4g02540 | DOF,WRKY,BHLH |
| At1g65010 | DOF,WRKY,BHLH |
| At3g45860 | DOF,WRKY,BHLH |
| At5g05160 | DOF,WRKY,BHLH |
| At2g46780 | DOF,WRKY,BHLH |
| At4g24050 | DOF,WRKY,BHLH |
| At5g63800 | DOF,WRKY,BHLH |
| At5g24610 | DOF,WRKY,BHLH |
| At1g17850 | DOF,WRKY,BHLH |
| At3g06380 | DOF,WRKY,BHLH |
| At1g12240 | DOF,WRKY,BHLH |
| At4g19160 | DOF,WRKY,BHLH |
| At3g59570 | DOF,WRKY,BHLH |
| At4g37450 | BHLH,AP2(CBF),HSF |
| At3g20090 | BHLH,AP2(CBF),HSF |
| At4g36670 | BHLH,AP2(CBF),HSF |
| At3g03190 | BHLH,AP2(CBF),HSF |
| At2g36145 | BHLH,AP2(CBF),HSF |
| At3g16090 | BHLH,AP2(CBF),HSF |
| At5g05440 | BHLH,AP2(CBF),HSF |
| At5g13690 | BHLH,AP2(CBF),HSF |
| At1g78270 | BHLH,AP2(CBF),HSF |
| At3g22680 | BHLH,AP2(CBF),HSF |
| At3g59090 | BHLH,AP2(CBF),HSF |
| At3g16240 | BHLH,AP2(CBF),HSF |
| At1g29440 | BHLH,AP2(CBF),HSF |
| At1g20560 | BHLH,AP2(CBF),HSF |
| At1g07020 | BHLH,AP2(CBF),HSF |
| At1g07000 | BHLH,AP2(CBF),HSF |
| At2g04230 | BHLH,AP2(CBF),HSF |
| At1g61100 | BHLH,AP2(CBF),HSF |
| At1g60670 | DOF |
| At2g47500 | DOF |
| At3g13620 | DOF |
| At1g12200 | DOF |
| At3g52540 | DOF |
| At5g62840 | DOF |
| At5g46780 | DOF |
| At5g46390 | DOF |
| At3g04940 | DOF |
| At4g37080 | DOF |
| At1g20900 | DOF |
| At1g74070 | DOF |
| At3g27060 | DOF |
| At4g14770 | DOF |
| At4g27300 | AP2(CBF),HSF |
| At3g09480 | AP2(CBF),HSF |
| At1g18560 | AP2(CBF),HSF |
| At1g05385 | AP2(CBF),HSF |
| At1g17140 | AP2(CBF),HSF |
| At1g08350 | AP2(CBF),HSF |
| At1g16820 | AP2(CBF),HSF |
| At5g26110 | AP2(CBF),HSF |
| At3g13070 | AP2(CBF),HSF |
| At5g20450 | AP2(CBF),HSF |
| At3g17470 | AP2(CBF),HSF |
| At1g67440 | AP2(CBF),HSF |
| At1g49750 | AP2(CBF),HSF |
| At3g01360 | AP2(CBF),HSF |
| At5g35480 | AP2(CBF),HSF |
| At1g21560 | AP2(CBF),HSF |
| At2g23430 | AP2(CBF),HSF |
| At3g06440 | AP2(CBF),HSF |
| At1g35350 | AP2(CBF),HSF |
| At4g22560 | AP2(CBF),HSF |
| At4g03820 | AP2(CBF),HSF |
| At4g10470 | AP2(CBF),HSF |
| At4g17540 | AP2(CBF),HSF |
| At2g44740 | AP2(CBF),HSF |
| At1g80790 | AP2(CBF),HSF |
| At4g14890 | AP2(CBF),HSF |
| At3g17020 | AP2(CBF),HSF |
| At2g01870 | AP2(CBF),HSF |
| At5g27710 | AP2(CBF),HSF |
| At4g09900 | AP2(CBF),HSF |
| At5g10320 | AP2(CBF),HSF |
| At4g12010 | AP2(CBF),HSF |
| At2g37480 | AP2(CBF),HSF |
| At4g19880 | WRKY |
| At1g29610 | WRKY |
| At1g69720 | WRKY |
| At2g31730 | WRKY |
| At5g25440 | WRKY |
| At5g65390 | WRKY |
| At1g05620 | WRKY |
| At5g61010 | WRKY |
| At5g55790 | WRKY |
| At3g57790 | WRKY |
| At1g03270 | WRKY |
| At1g74100 | WRKY |
| At3g30180 | WRKY |
| At3g01660 | WRKY |
| At1g52540 | WRKY |
| At4g10550 | WRKY |
| At5g13140 | WRKY |
| At3g02630 | WRKY |
| At4g01610 | WRKY |
| At5g24160 | WRKY |
| At3g49870 | WRKY |
| At1g49180 | WRKY |
| At2g42490 | WRKY |
| At5g56380 | WRKY |
| At5g61420 | WRKY |
| At4g10330 | WRKY |
| At3g05100 | WRKY |
| At1g55090 | BHLH,AP2(CBF) |
| At5g51820 | BHLH,AP2(CBF) |
| At2g41870 | BHLH,AP2(CBF) |
| At1g18590 | BHLH,AP2(CBF) |
| At4g33460 | BHLH,AP2(CBF) |
| At1g17620 | BHLH,AP2(CBF) |
| At4g25940 | BHLH,AP2(CBF) |
| At3g12690 | BHLH,AP2(CBF) |
| At1g49730 | BHLH,AP2(CBF) |
| At2g18050 | BHLH,AP2(CBF) |
| At1g55020 | BHLH,AP2(CBF) |
| At2g02780 | BHLH,AP2(CBF) |
| At4g37980 | BHLH,AP2(CBF) |
| At2g30280 | BHLH,AP2(CBF) |
| At4g30520 | BHLH,AP2(CBF) |
| At5g45020 | BHLH,AP2(CBF) |
| At3g01370 | BHLH,AP2(CBF) |
| At3g26490 | BHLH,AP2(CBF) |
| At4g15820 | BHLH,AP2(CBF) |
| At1g52340 | BHLH,AP2(CBF) |
| At1g09750 | BHLH,AP2(CBF) |
| At3g45230 | BHLH,AP2(CBF) |
| At3g28130 | BHLH,AP2(CBF) |
| At4g13770 | BHLH,AP2(CBF) |
| At3g56840 | BHLH,AP2(CBF) |
| At2g34470 | BHLH,AP2(CBF) |
| At1g60010 | BHLH,AP2(CBF) |
| At5g57785 | BHLH,AP2(CBF) |
| At5g39080 | BHLH,AP2(CBF) |
| At3g46670 | DOF,BHLH,AP2(CBF),HSF |
| At3g15420 | DOF,BHLH,AP2(CBF),HSF |
| At5g63190 | DOF,BHLH,AP2(CBF),HSF |
| At3g11840 | DOF,BHLH,AP2(CBF),HSF |
| At4g31920 | DOF,BHLH,AP2(CBF),HSF |
| At4g34160 | DOF,BHLH,AP2(CBF),HSF |
| At3g49060 | DOF,BHLH,AP2(CBF),HSF |
| At2g01910 | DOF,BHLH,AP2(CBF),HSF |
| At5g41400 | DOF,BHLH,AP2(CBF),HSF |
| At4g20780 | DOF,BHLH,AP2(CBF),HSF |
| At3g22420 | DOF,BHLH,AP2(CBF),HSF |
| At1g76890 | DOF,BHLH,AP2(CBF),HSF |
| At2g47930 | DOF,BHLH,AP2(CBF),HSF |
| At4g21870 | DOF,BHLH,AP2(CBF),HSF |
| At4g27450 | DOF,BHLH,AP2(CBF),HSF |
| At4g16140 | DOF,BHLH,AP2(CBF),HSF |
| At2g46250 | DOF,BHLH,AP2(CBF),HSF |
| At1g10020 | DOF,BHLH,AP2(CBF),HSF |
| At5g64370 | DOF,BHLH,AP2(CBF),HSF |
| At5g14220 | DOF,WRKY,HSF |
| At5g39360 | DOF,WRKY,HSF |
| At4g19380 | DOF,WRKY,HSF |
| At1g76090 | DOF,WRKY,HSF |
| At5g65490 | DOF,WRKY,HSF |
| At5g09300 | DOF,WRKY,HSF |
| At1g27210 | DOF,WRKY,HSF |
| At2g31120 | DOF,WRKY,HSF |
| At1g51280 | DOF,WRKY,HSF |
| At1g78110 | DOF,WRKY,HSF |
| At3g50880 | DOF,WRKY,HSF |
| At5g17880 | DOF,WRKY,HSF |
| At1g74510 | DOF,WRKY,HSF |
| At1g11310 | DOF,WRKY,HSF |
| At5g44860 | DOF,WRKY,HSF |
| At1g35710 | DOF,WRKY,HSF |
| At1g11050 | DOF,WRKY,HSF |
| At2g30080 | DOF,WRKY,HSF |
| At3g45850 | DOF,WRKY,HSF |
| At2g22650 | DOF,WRKY,HSF |
| At2g26300 | DOF,WRKY,HSF |
| At2g35155 | DOF,WRKY,HSF |
| At3g23590 | DOF,WRKY,HSF |
| At5g01030 | DOF,WRKY,HSF |
| At1g09390 | DOF,WRKY,HSF |
| At3g50650 | DOF,WRKY,HSF |
| At5g27350 | DOF,WRKY,HSF |
| At4g13510 | DOF,WRKY,HSF |
| At3g61670 | DOF,WRKY,HSF |
| At3g02180 | DOF,WRKY,HSF |
| At4g15800 | DOF,WRKY,HSF |
| At3g09980 | DOF,WRKY,HSF |
| At2g25590 | DOF,WRKY,HSF |
| At1g51950 | DOF,WRKY,HSF |
| At1g20640 | DOF,WRKY,HSF |
| At5g03380 | DOF,WRKY,HSF |
| At3g05230 | DOF,WRKY,HSF |
| At3g28180 | DOF,WRKY,HSF |
| At5g48730 | DOF,WRKY,HSF |
| At2g44500 | DOF,WRKY,HSF |
| At4g39190 | DOF,WRKY,HSF |
| At1g53230 | DOF,WRKY,HSF |
| At4g28100 | DOF,WRKY,HSF |
| At1g30530 | DOF,WRKY,HSF |
| At5g49960 | WRKY,BHLH,AP2(CBF) |
| At1g17350 | WRKY,BHLH,AP2(CBF) |
| At1g22360 | WRKY,BHLH,AP2(CBF) |
| At5g15770 | WRKY,BHLH,AP2(CBF) |
| At5g28500 | WRKY,BHLH,AP2(CBF) |
| At4g19510 | WRKY,BHLH,AP2(CBF) |
| At1g69370 | WRKY,BHLH,AP2(CBF) |
| At3g29230 | WRKY,BHLH,AP2(CBF) |
| At4g30825 | WRKY,BHLH,AP2(CBF) |
| At3g13226 | WRKY,BHLH,AP2(CBF) |
| At3g59050 | WRKY,BHLH,AP2(CBF) |
| At3g43300 | WRKY,BHLH,AP2(CBF) |
| At5g66250 | WRKY,BHLH,AP2(CBF) |
| At4g38850 | WRKY,BHLH,AP2(CBF) |
| At5g20110 | WRKY,BHLH,AP2(CBF) |
| At1g52720 | WRKY,BHLH,AP2(CBF) |
| At1g31480 | DOF,WRKY,BHLH,HSF |
| At2g35820 | DOF,WRKY,BHLH,HSF |
| At5g60400 | DOF,WRKY,BHLH,HSF |
| At5g40910 | DOF,WRKY,BHLH,HSF |
| At5g35750 | DOF,WRKY,BHLH,HSF |
| At5g58787 | DOF,WRKY,BHLH,HSF |
| At2g33170 | DOF,WRKY,BHLH,HSF |
| At1g12330 | DOF,WRKY,BHLH,HSF |
| At2g17500 | DOF,WRKY,BHLH,HSF |
| At5g03610 | DOF,WRKY,BHLH,HSF |
| At3g18930 | DOF,WRKY,BHLH,HSF |
| At1g22050 | DOF,WRKY,BHLH,HSF |
| At5g43070 | DOF,WRKY,BHLH,HSF |
| At4g34770 | DOF,WRKY,BHLH,HSF |
| At1g22430 | DOF,WRKY,BHLH,HSF |
| At4g27800 | DOF,WRKY,BHLH,HSF |
| At1g20330 | DOF,WRKY,BHLH,HSF |
| At4g14680 | DOF,WRKY,BHLH,HSF |
| At1g63180 | DOF,WRKY,BHLH,HSF |
| At1g53390 | DOF,WRKY,BHLH,HSF |
| At1g60550 | DOF,WRKY,BHLH,HSF |
| At4g31000 | DOF,WRKY,BHLH,HSF |
| At1g55850 | DOF,WRKY,BHLH,HSF |
| At1g22400 | DOF,WRKY,BHLH,HSF |
| At5g64240 | DOF,WRKY,BHLH,HSF |
| At5g53880 | DOF,WRKY,BHLH,HSF |
| At1g11080 | DOF,BZIP,AP2(CBF) |
| At2g45740 | DOF,BZIP,AP2(CBF) |
| At5g58570 | DOF,BZIP,AP2(CBF) |
| At5g63180 | DOF,BZIP,AP2(CBF) |
| At2g34620 | DOF,BZIP,AP2(CBF) |
| At4g22730 | DOF,BZIP,BHLH,HSF |
| At3g05750 | DOF,BZIP,BHLH,HSF |
| At1g74300 | DOF,BZIP,BHLH,HSF |
| At1g31230 | DOF,BZIP,BHLH,HSF |
| At1g53560 | DOF,BZIP,BHLH,HSF |
| At5g16720 | DOF,BHLH,AP2(CBF) |
| At1g80290 | DOF,BHLH,AP2(CBF) |
| At1g06830 | DOF,BHLH,AP2(CBF) |
| At3g53170 | DOF,BHLH,AP2(CBF) |
| At2g35780 | DOF,BHLH,AP2(CBF) |
| At4g35920 | DOF,BHLH,AP2(CBF) |
| At1g29510 | DOF,BHLH,AP2(CBF) |
| At4g38350 | DOF,BHLH,AP2(CBF) |
| At3g18290 | DOF,BHLH,AP2(CBF) |
| At5g04530 | DOF,BHLH,AP2(CBF) |
| At1g11720 | DOF,BHLH,AP2(CBF) |
| At3g44970 | DOF,BHLH,AP2(CBF) |
| At3g01470 | DOF,BHLH,AP2(CBF) |
| At4g11410 | DOF,BHLH,AP2(CBF) |
| At5g53170 | DOF,BHLH,AP2(CBF) |
| At1g73655 | DOF,BHLH,AP2(CBF) |
| At2g24180 | DOF,BHLH,AP2(CBF) |
| At3g10870 | DOF,BHLH,AP2(CBF) |
| At2g21185 | DOF,BHLH,AP2(CBF) |
| At3g02300 | DOF,BHLH,AP2(CBF) |
| At4g14440 | DOF,BHLH,AP2(CBF) |
| At1g47960 | DOF,BHLH,AP2(CBF) |
| At3g12560 | DOF,BHLH,AP2(CBF) |
| At1g80630 | DOF,BHLH,AP2(CBF) |
| At2g41820 | DOF,BHLH,AP2(CBF) |
| At5g01710 | DOF,BHLH,AP2(CBF) |
| At1g76530 | DOF,MYB,BHLH |
| At5g27360 | DOF,MYB,BHLH |
| At1g67830 | DOF,MYB,BHLH |
| At1g78890 | DOF,MYB,BHLH |
| At5g26600 | DOF,MYB,BHLH |
| At3g46540 | DOF,MYB,BHLH |
| At1g67040 | DOF,MYB,BHLH |
| At1g68780 | DOF,MYB,BHLH |
| At5g03460 | DOF,MYB,BHLH |
| At2g24630 | DOF,MYB,BHLH |
| At5g04360 | DOF,MYB,BHLH |
| At1g34640 | DOF,MYB,BHLH |
| At5g13730 | DOF,MYB,BHLH |
| At2g16380 | DOF,MYB,BHLH |
| At1g70280 | DOF,MYB,BHLH |
| At1g49480 | DOF,MYB,BHLH |
| At4g33550 | DOF,MYB |
| At2g41940 | DOF,MYB |
| At5g47730 | DOF,MYB |
| At5g58310 | DOF,MYB |
| At1g04000 | DOF,MYB |
| At2g25830 | DOF,MYB |
| At2g04160 | MADS |
| At5g67290 | MADS |
| At2g39890 | MADS |
| At4g20840 | MADS |
| At5g16870 | MADS |
| At1g53350 | MYB |
| At5g40330 | MYB |
| At1g68600 | MYB |
| At4g19985 | MYB |
| At4g24790 | MYB |
| At5g53830 | MYB |
| At3g25560 | MYB |
| At4g14510 | MYB |
| At5g03430 | MYB |
| At1g09010 | MYB |
| At1g23400 | MYB |
| At1g78180 | MYB |
| At5g52540 | MYB |
| At1g27480 | MYB |
| At1g72030 | MYB |
| At5g01090 | MYB |
| At5g61190 | DOF,BZIP,HSF |
| At3g48610 | DOF,BZIP,HSF |
| At3g08600 | DOF,BZIP,HSF |
| At3g54260 | DOF,BZIP,HSF |
| At3g05430 | DOF,BZIP,HSF |
| At5g53450 | DOF,BZIP,HSF |
| At3g46790 | DOF,BHLH,MADS |
| At3g18710 | DOF,BHLH,MADS |
| At3g53920 | DOF,BHLH,MADS |
| At3g43800 | DOF,BHLH,MADS |
| At5g67070 | DOF,BHLH,MADS |
| At2g15830 | DOF,BHLH,MADS |
| At5g47640 | DOF,BHLH,MADS |
| At4g35550 | DOF,MYB,MADS |
| At2g27920 | DOF,MYB,MADS |
| At5g19310 | DOF,MYB,MADS |
| At2g28940 | WRKY,BZIP,HSF |
| At1g19670 | WRKY,BZIP,HSF |
| At4g37480 | WRKY,BZIP,HSF |
| At2g01420 | DOF,MYB,BHLH, |
| At4g10270 | DOF,MYB,BHLH, |
| At1g80050 | DOF,MADS |
| At5g47900 | DOF,MADS |
| At5g34850 | DOF,MYB,AP2(CBF) |
| At1g48330 | DOF,MYB,AP2(CBF) |
| At1g58150 | DOF,MYB,AP2(CBF) |
| At3g09580 | DOF,MYB,AP2(CBF) |
| At1g59620 | BHLH,MADS |
| At5g04810 | BHLH,MADS |
| At2g39950 | BHLH,MADS |
| At2g37790 | BHLH,MADS |
| At2g20340 | MYB,BHLH |
| At4g25780 | MYB,BHLH |
| At4g35880 | MYB,BHLH |
| At1g29460 | MYB,BHLH |
| At1g64850 | MYB,BHLH |
| At2g44230 | MYB,BHLH |
| At5g42620 | MYB,BHLH |
| At5g28020 | MYB,BHLH |
| At1g29450 | MYB,BHLH |
| At1g14030 | MYB,BHLH |
| At2g28470 | MYB,BHLH |
| At2g42190 | MYB,BHLH |
| At2g37950 | MYB,BHLH |
| At5g45060 | BHLH,TCP2 |
| At5g50020 | BHLH,TCP2 |
| At1g54050 | BZIP |
| At5g16980 | BZIP |
| At3g28430 | BZIP |
| At3g02050 | BZIP |
| At5g16180 | BZIP |
| At2g04690 | BZIP |
| At3g53800 | BZIP |
| At4g03150 | BZIP |
| At1g31920 | BHLH,MADS,AP2(CBF) |
| At5g05480 | BHLH,MADS,AP2(CBF) |
| At1g62800 | BZIP,BHLH,AP2(CBF) |
| At2g21950 | BZIP,BHLH,AP2(CBF) |
| At4g04830 | BZIP,BHLH,AP2(CBF) |
| At1g68190 | BZIP,BHLH,AP2(CBF) |
| At2g40970 | BZIP,BHLH,AP2(CBF) |
| At1g52770 | MYB |
| At1g18810 | MYB |
| At5g39210 | BHLH,TCP2 |
| At1g51140 | BHLH,TCP2 |
| At3g45050 | BHLH,TCP2 |
| At4g38660 | BHLH,TCP2 |
| At5g45800 | DOF,BZIP |
| At5g18690 | DOF,BZIP |
| **Additional file 3**. Down-regulated Genes and their predicted regulators. a: Locus numbers of the genes that are the putative targets, b: TF families predicted to regulate the genes in a. | |
